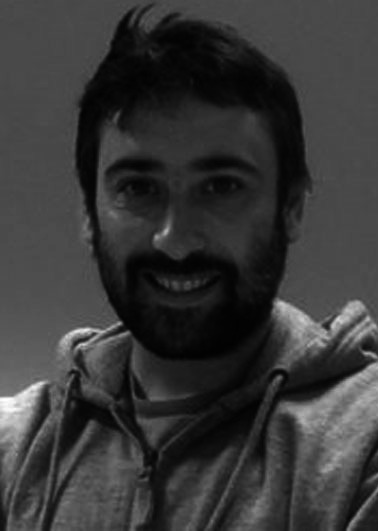# (Hetero)aryl‐S^VI^ Fluorides: Synthetic Development and Opportunities

**DOI:** 10.1002/anie.202200904

**Published:** 2022-04-27

**Authors:** Marc Magre, Shengyang Ni, Josep Cornella

**Affiliations:** ^1^ Max-Planck-Institut für Kohlenforschung Kaiser-Wilhelm-Platz 1 45470 Mülheim an der Ruhr Germany

**Keywords:** Fluorine, Pentafluorosulfanyl Arenes, Sulfinyl Trifluorides, Sulfur, Tetrafluorosulfanyl Chlorides

## Abstract

(Hetero)arylsulfur compounds where the S atom is in the oxidation state VI represent a large percentage of the molecular functionalities present in organic chemistry. More specifically, (hetero)aryl‐S^VI^ fluorides have recently received enormous attention because of their potential as chemical biology probes, as a result of their reactivity in a simple, modular, and efficient manner. Whereas the synthesis and application of the level 1 fluorination at S^VI^ atoms (sulfonyl and sulfonimidoyl fluorides) have been widely studied and reviewed, the synthetic strategies towards higher levels of fluorination (levels 2 to 5) are somewhat more limited. This Minireview evaluates and summarizes the progress in the synthesis of highly fluorinated aryl‐S^VI^ compounds at all levels, discussing synthetic strategies, reactivity, the advantages and disadvantages of the synthetic procedures, the proposed mechanisms, and the potential upcoming opportunities.

## Graphical abstract

The number of methods that exploit the synthesis of (hetero)arylsulfur(VI) fluorides is steadily growing due to their potential in organic synthesis. This Minireview covers the synthesis of arylsulfur(VI) fluorides from levels 2 to 5 of fluorination, the substrate scope, mechanisms, and the further applicability of these fluorinated compounds.

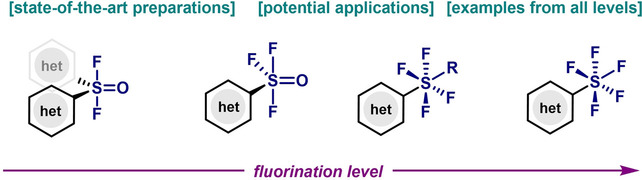

## 1 Introduction

**Figure 1:**
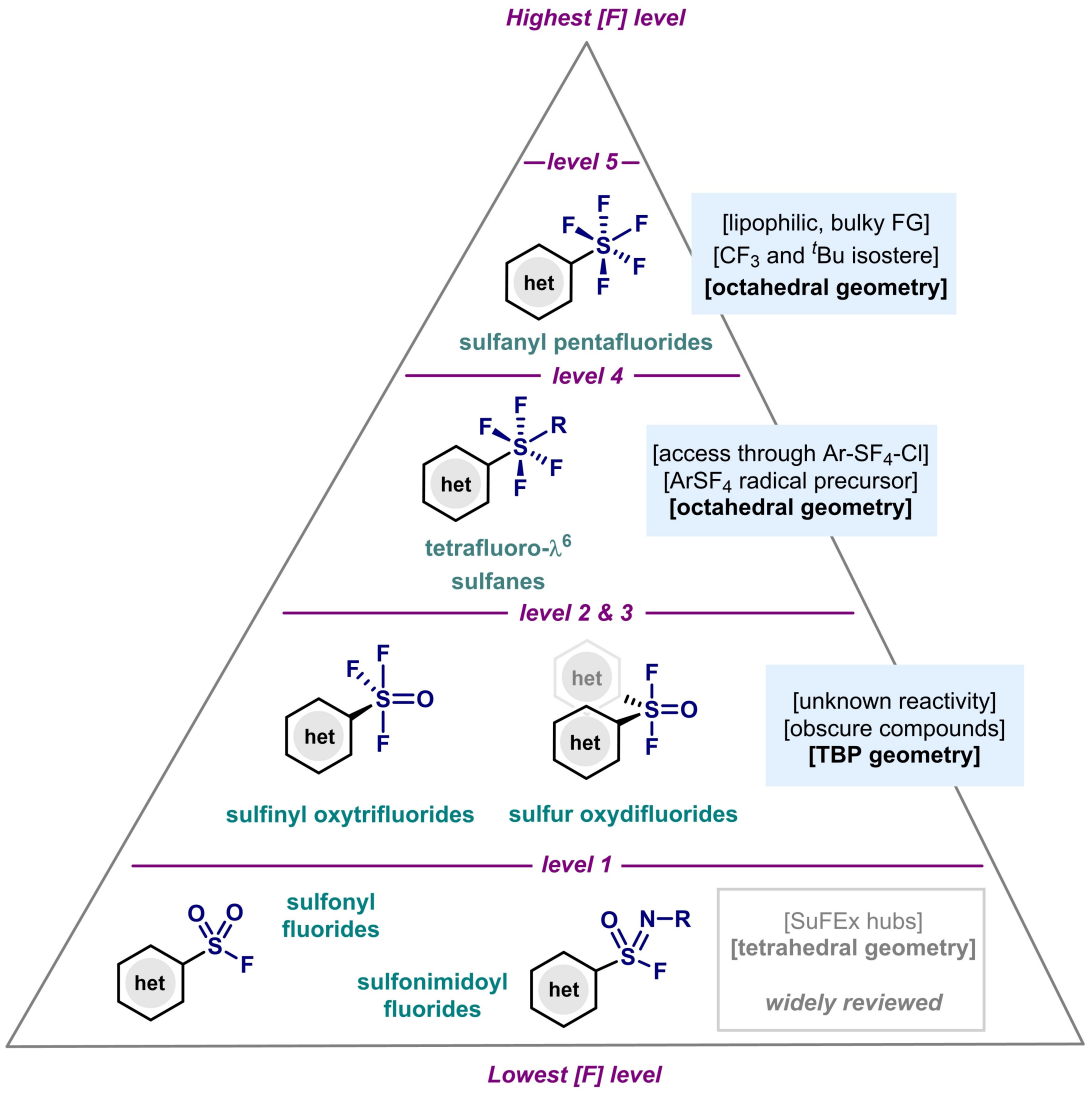
Fluorinated arylsulfur(VI) compounds.

Aryl‐S^VI^ compounds (including also heteroaryl ones) are ever‐present functionalities in organic synthesis, spanning from the well‐known arylsulfonyl chloride electrophiles^[1]^ to the aryl sulfonamides that are widely present in many pharmaceuticals and agrochemicals.^[2]^ Indeed, the synthesis and applications of these compounds has been widely reviewed and their synthesis is common textbook knowledge.^[3]^ Within the group of aryl‐S^VI^ compounds, however, there is a class of compounds that has received comparatively less attention: these are aryl‐S^VI^ fluorides, where the S^VI^ center is directly attached to F atoms. Indeed, compounds of this class have recently been re‐evaluated, as they have shown interesting properties and applications in various fields of expertise.^[4]^ For example, arylsulfonyl fluorides^[5]^ and arylsulfonimidoyl fluorides^[6]^ have been studied in sulfur–fluoride exchange reactions (SuFEx), and their use as chemical probes for chemical biology has proved highly valuable.^[7]^ However, from a structural and molecular point of view, arylsulfonyl and arylsulfonimidoyl fluorides both represent the lowest level of fluorination at the S atom, with only one fluorine atom attached to the tetrahedral S atom (level 1, Figure 1). Despite the success of level 1,^[8]^ compounds with a S atom at higher levels of fluorination are less well known, but still accessible. For example, diarylsulfur oxide difluorides (level 2) and arylsulfinyl trifluorides (level 3), which present a sulfur atom with a trigonal bypyramidal (TBP) geometry, have been the least studied of the fluorinated aryl‐S^VI^ compounds, with untapped reactivity and applications. Moving up the pyramid, one can find aryltetrafluoro‐λ^6^‐sulfanyl chlorides and aryltetrafluoro‐λ^6^‐sulfanes, with various synthetic procedures reported in the literature for their preparation. In particular, aryltetrafluoro‐λ^6^‐sulfanyl chlorides have been widely studied and applied as precursors for pentafluoro(aryl)‐λ^6^‐sulfane (Ar−SF_5_) and deoxyfluorinating agents.^[9]^ In contrast to the lower levels of fluorination, the S atom at level 4 presents an octahedral geometry, which leads to potential structural isomerism. Finally, the highest level (level 5) is occupied by pentafluoro(aryl)‐λ^6^‐sulfanylarenes (Ar−SF_5_), which recently gained the attention of the chemistry community because of the unique properties of the SF_5_ group in material science and medicinal chemistry fields.^[10]^ With an octahedral geometry around the S center and a square‐pyramidal array of fluorine atoms, the symmetrical SF_5_ group is sterically highly demanding, thus it can be considered as an isostere of the *tert‐*butyl (^*t*^Bu) and the trifluoromethyl (CF_3_) groups.[^11^, ^12^]

In this Minireview, the synthetic methods, substrate scope, mechanism, and applications of levels 2 to 5 will be evaluated and summarized. Compounds belonging to level 1 (Figure 1) have already been extensively reviewed and will not be included.^[8]^ The organization of this Minireview will follow an increase in the fluorination level, and the different synthetic procedures will be presented and discussed chronologically.

## 2 Fluorination Level 2

**Scheme 1:**
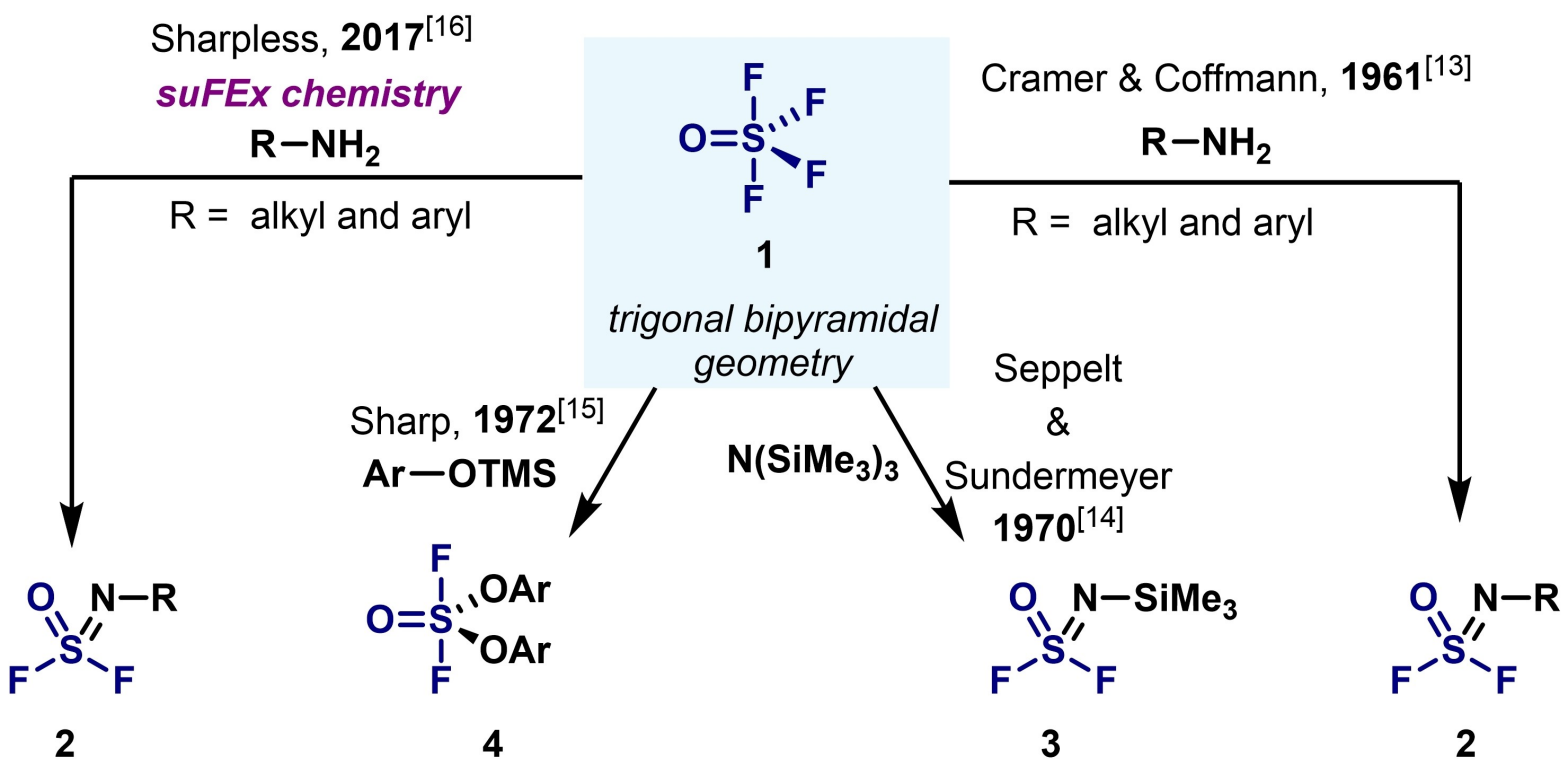
Use of sulfur(VI) fluoride (SOF_4_) gas as a SuFEx reagent.

In 1961, Cramer and Coffman reported the reaction of gaseous thionyl tetrafluoride (**1**, SOF_4_) with primary amines leading to iminosulfur oxydifluorides **2** in good yields (Scheme 1).^[13]^ This seminal example set a foundation stone for further applications, such as those of Seppelt and Sundermeyer as well as Sharp and co‐workers,[^14^, ^15^] who capitalized on the strong Si−F bond to react silylated nucleophiles with SOF_4_. This field, however, remained dormant until Sharpless and co‐workers realized the balanced reactivity and stability of iminosulfur oxydifluorides **2**, and their value in organic synthesis and chemical biology (Scheme 1).^[16]^

**Scheme 2:**
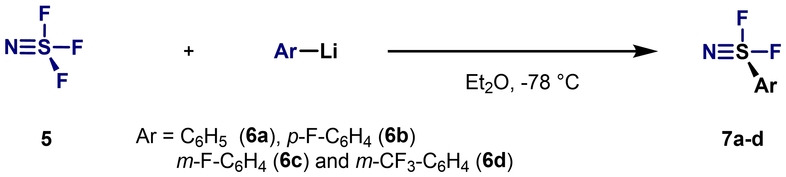
Synthesis of ArSNF_2_ by Clifford et al.^[17]^

In 1978, Clifford et al. reported the synthesis of aryl‐S^VI^ difluorides **7** by reacting thiazyl trifluoride (SNF_3_; **5**) with various aryllithium compounds **6** at −78 °C (Scheme 2).^[17]^ NMR spectroscopy studies revealed that the ‐SNF_2_ group is more strongly electron‐withdrawing than the ‐NO_2_ and ‐SF_5_ groups.

**Scheme 3:**
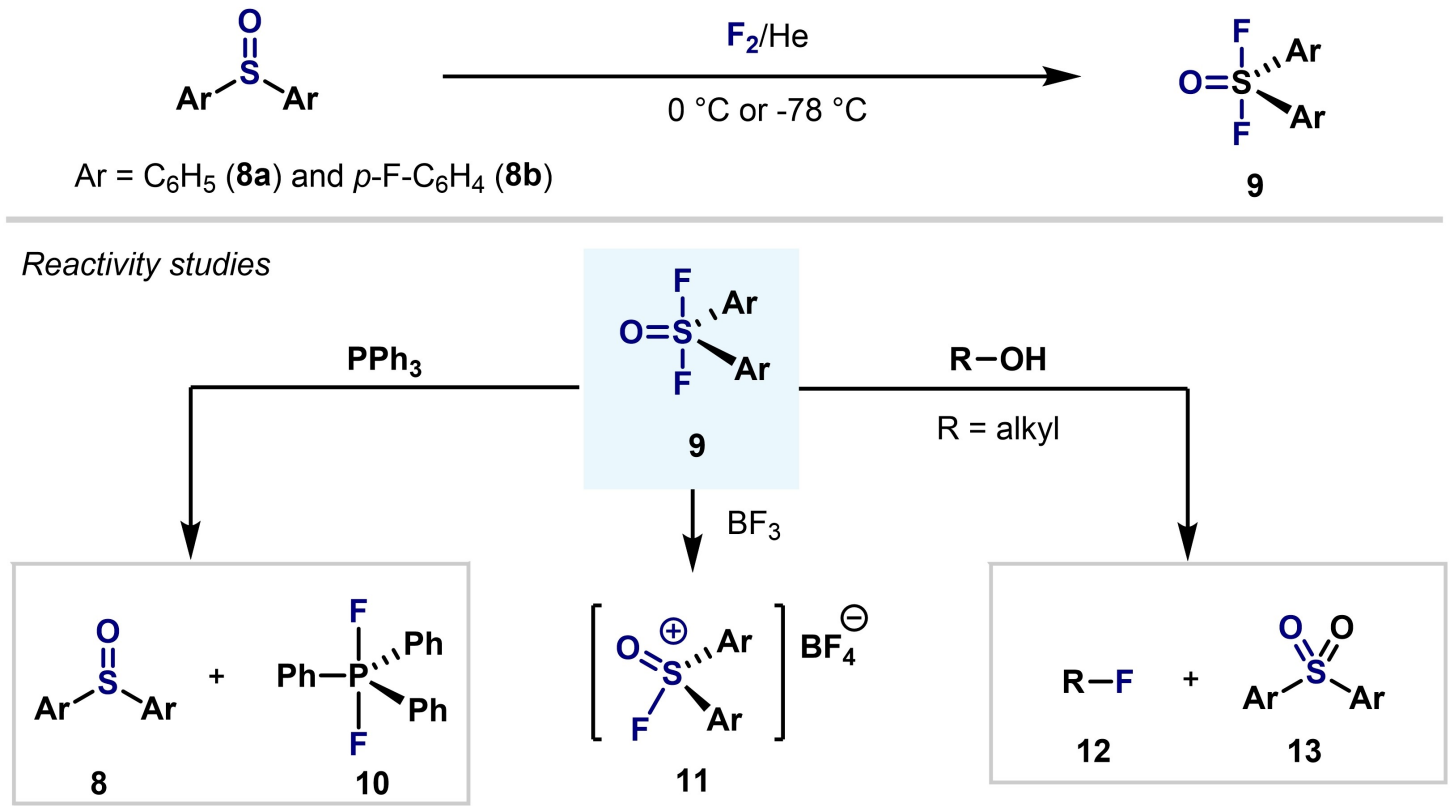
Synthesis and reactivity studies of Ar_2_SOF_2_ by Ruppert.^[18]^

A year later, Ruppert reported the first examples of diarylsulfur(VI) oxide difluorides.^[18]^ Diaryl sulfoxides **8** could be fluorinated directly by liquid‐phase fluorination using fluorine (F_2_) at low temperatures (Scheme 3). This synthetic procedure allowed, for the first time, access to diarylsulfur(VI) oxide difluorides **9**, which cannot be obtained by the direct arylation of SOF_4_ (**1**). The use of other strong fluorinating agents, such as XeF_2_, failed as alternatives for direct difluorination. The reactivity of the newly synthesized diaryl‐S^VI^ difluorides **9** was also evaluated: they are highly sensitive to moisture and can only be handled under dried protective gas. During these reactivity studies it was found that the Ar_2_S(O)F_2_ compounds display high activity as fluorinating agents. Interestingly, in the presence of BF_3_, oxo‐cationic species **11** were obtained.

**Scheme 4:**
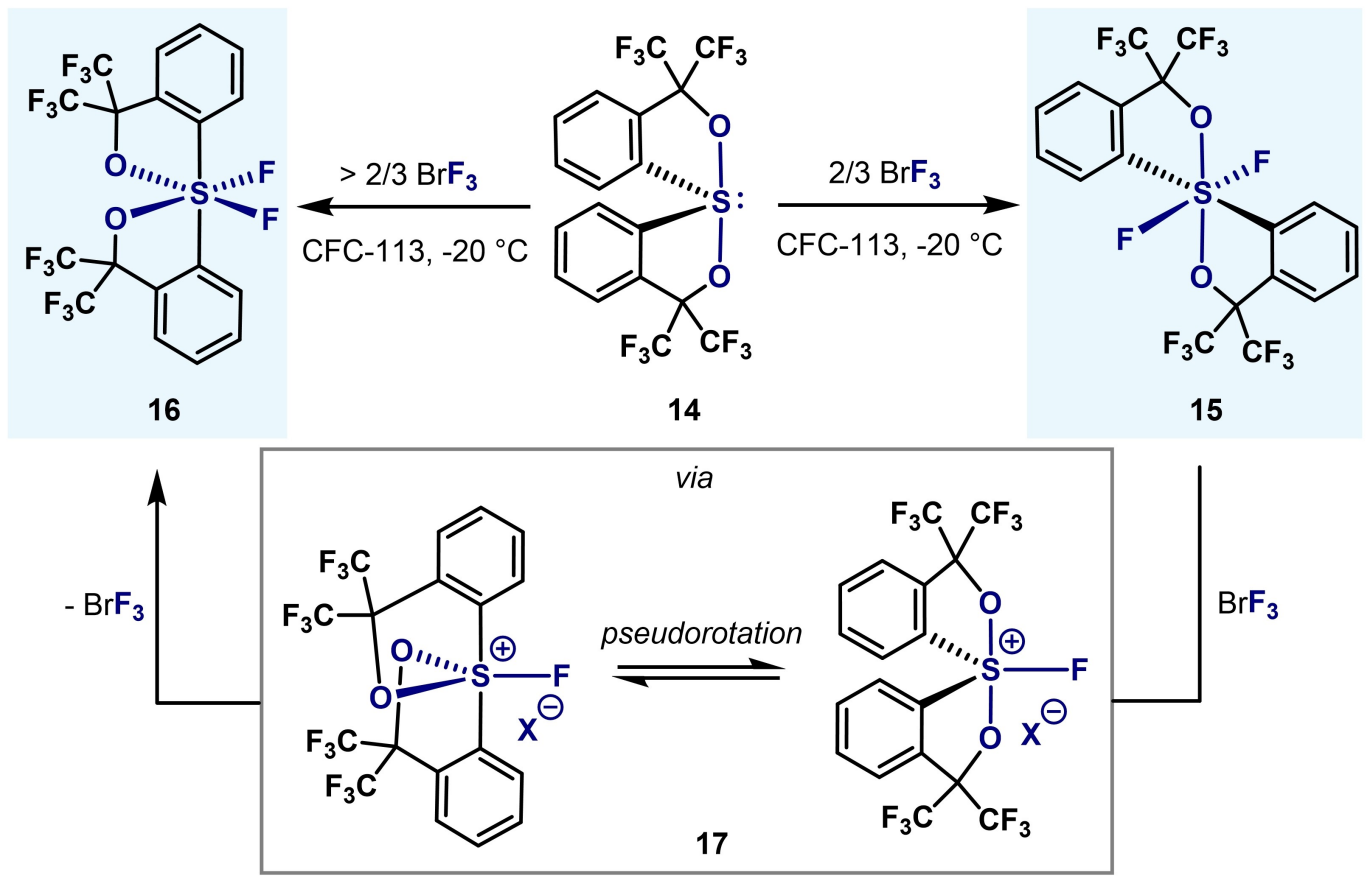
Synthesis of *cis*‐ and *trans*‐difluoropersulfuranes by Michalak and Martin.[^19^, ^20^]

Michalak and Martin reported that sulfurane **14** can be oxidatively fluorinated using an equimolar amount of BrF_3_ as the difluorinating agent, thereby obtaining *all‐trans* difluoride **15** (Scheme 4).^[19]^ Surprisingly, when the same difluorination was studied using an excess of BrF_3_, the *cis*‐isomer **16** was obtained. Both isomers exhibited the same reactivity towards hydrolysis (highly reactive). Structures **15** and **16** were confirmed by means of NMR spectroscopy and single‐crystal XRD.^[20]^ Whereas heating a solution of **15** did not show isomerization to **16**, the presence of catalytic amounts of the Lewis acid SbF_5_ afforded *cis*‐isomer **16** quantitatively, presumably via a persulfonium salt intermediate **17**. Thus, it was hypothesized that the formation of **16**, rather than **15**, in the oxidation of the sulfurane **14** with excess of BrF_3_ arises from the Lewis acidity of BrF_3_ present in solution leading to the formation of the persulfonium intermediate **17**, which leads to isomer **16** after pseudorotation and fluoride capture.

**Scheme 5:**
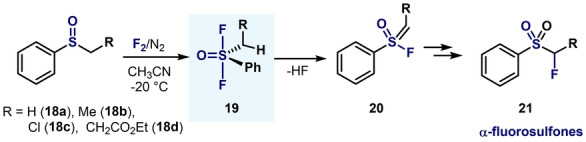
Synthesis of α‐fluorosulfones via aryl‐alkyl‐sulfur(VI) difluoride.^[21]^

In 1995, Kaneko and co‐workers capitalized on Ruppert′s methodology for the synthesis of α‐fluorosulfones (Scheme 5). Interestingly, when aryl‐alkyl sulfoxides **18** were treated with F_2_/N_2_, S^VI^ difluoride species **19** were observed by ^19^F NMR spectroscopy, and these species readily evolved into the dehydrofluorinated products **20**, by virtue of the loss of HF.^[21]^

**Scheme 6:**
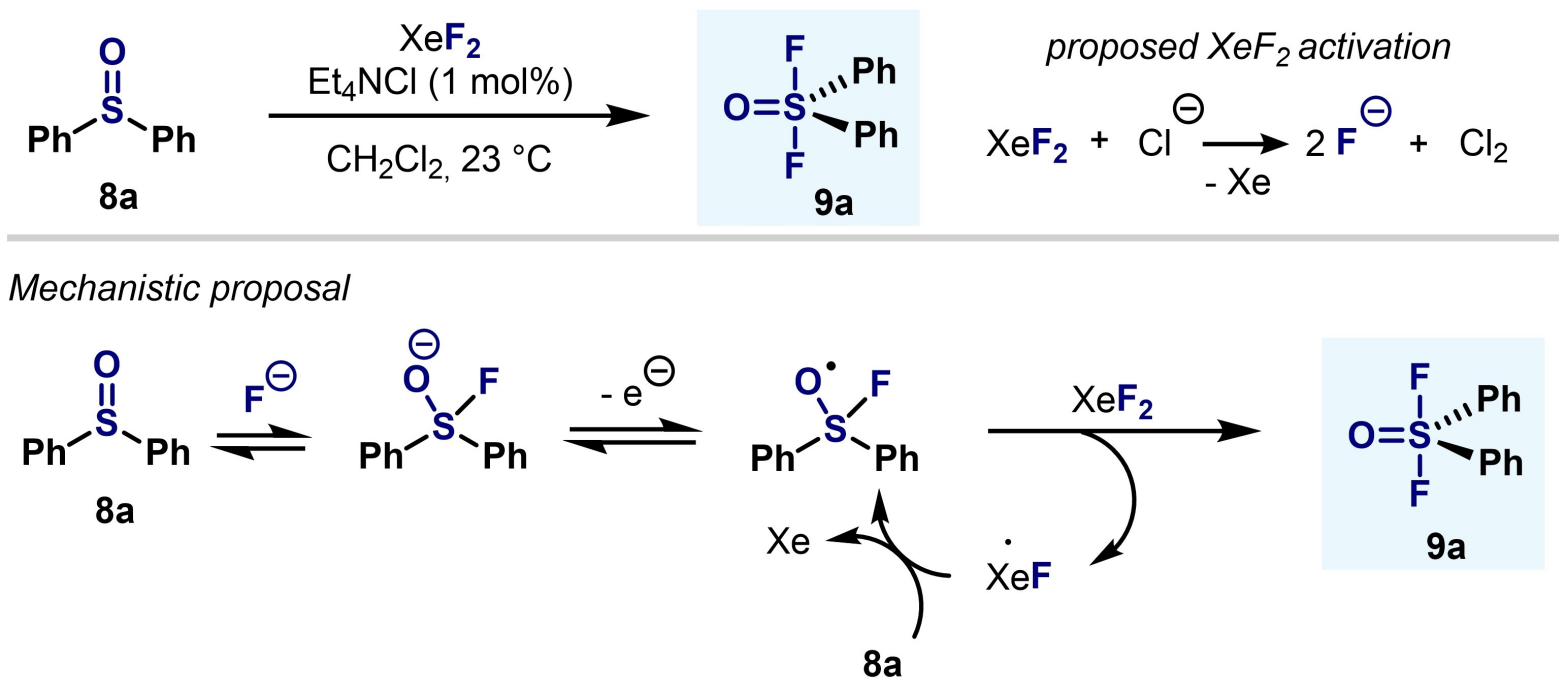
XeF_2_/Cl^−^ system: synthesis of Ar_2_SOF_2_ reported by Janzen and Ou.[^22^, ^23^]

A breakthrough in the synthesis of aryl‐S^VI^ difluorides was made in the same year by Janzen and Ou (Scheme 6). Oxidative fluorination of aryl‐S(IV) compounds such as diphenyl sulfoxide or diphenylsulfur difluoride occurs under mild conditions in the presence of XeF_2_ and catalytic amounts of chloride anions.^[22]^ In this manner, diarylsulfur(VI) difluorides were obtained in quantitative yields and in short reaction times. Mechanistically, it was postulated that a Cl‐mediated activation of XeF_2_ for the oxidative fluorination of diarylsulfoxides would initiate the reaction, which would be followed by a radical chain reaction propagated by Ph_2_S(O⋅)F species.^[23]^

**Scheme 7:**
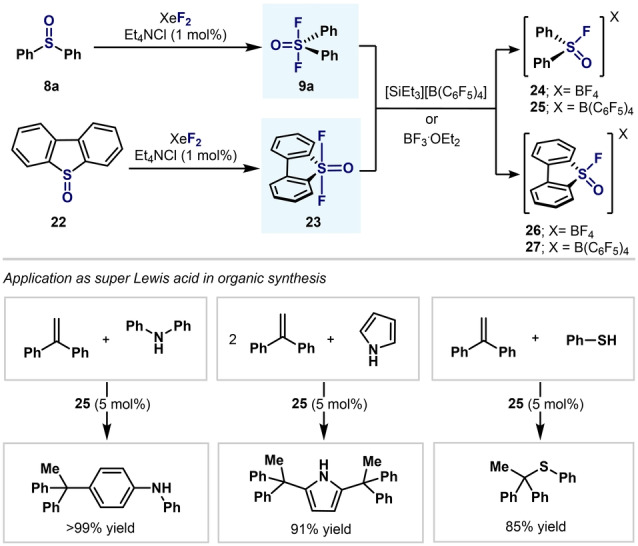
Synthesis and application of diarylfluorosulfoxonium cations by Stephan and co‐workers.^[24]^

In 2016, Stephan and co‐workers developed a variety of diaryl fluorosulfoxonium cations (**24**–**27**) and applied them to several Lewis acid catalyzed reactions (Scheme 7).^[24]^ Synthetically inspired by the procedure developed by Janzen and Ou, Stephan and co‐workers were able to oxidize diaryl sulfoxides **8** **a** and **22** to their corresponding diarylsulfur(VI) difluorides in excellent yields and, after fluoride abstraction with either BF_3_ or [SiEt_3_][B(C_6_F_5_)_4_], fluorosulfoxonium cations **24**–**27** could be isolated and characterized by NMR spectroscopy and single‐crystal XRD. Experimental analysis (Gutmann–Beckett method) and theoretical calculations (DFT) confirmed the high Lewis acidity of fluorosulfoxonium cations **24**–**27**. Finally, compound **25** was demonstrated to be highly active in Friedel–Crafts‐type reactions with 1,1‐diphenylethene (Scheme 7, bottom).

**Scheme 8:**
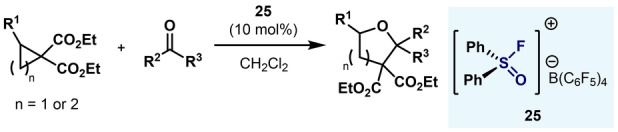
Application of diarylfluorosulfoxonium cation **25** in annulation reactions by Panossian and co‐workers.^[25]^

Similarly, in 2021 Panossian and co‐workers applied Lewis acidic sulfoxonium cation **25** in ring‐opening [3+2] and [4+2] annulations (Scheme 8). Excellent yields for a wide substrate scope were obtained, thus validating the excellent Lewis acid character of sulfoxonium cation **25**.^[25]^

## 3 Fluorination Level 3

**Scheme 9:**
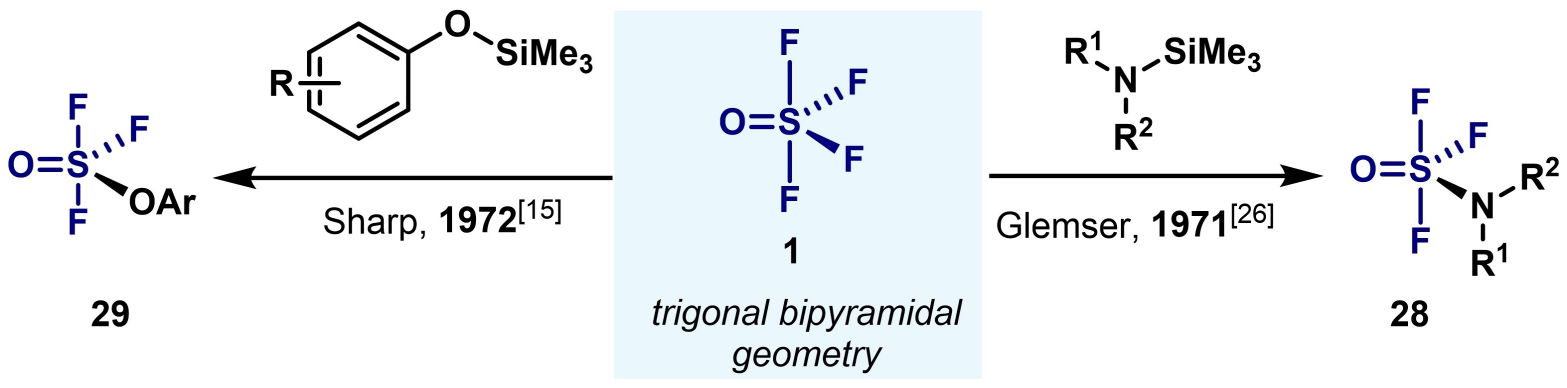
Pioneering syntheses of sulfur(VI) trifluorides.

Aryl‐S^VI^ trifluorides are the least studied of the fluorination levels known, with few examples described in the literature. To place their development into context, it is important to mention the seminal studies by the groups of Glemser and Sharp in 1971 and 1972: SOF_4_ (**1**) can react with trimethylsilylamines^[26]^ and trimethylsilyl aryl ethers^[15]^ to afford the corresponding monosubstituted sulfur(VI) trifluorides **28** and **29** (Scheme 9).

**Scheme 10:**
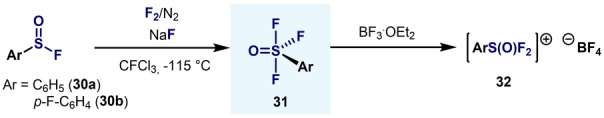
Synthesis of aryl‐S^VI^ oxytrifluorides by Ruppert.^[27]^

Similar to aryl‐S^VI^ difluorides, the direct monoarylation of SOF_4_ (**1**) is not synthetically viable and, therefore, alternative synthetic routes were required. In 1980, Ruppert showed that aryl‐S^VI^ oxytrifluorides **31** could be obtained by direct fluorine addition to sulfinic fluorides **30** using F_2_ at very low reaction temperatures.^[27]^ ^19^F NMR spectroscopy confirmed the finding through the presence of an AX_2_ pattern, which is in agreement with a trigonal bipyramidal geometry of an S atom with two fluorine atoms occupying the axial positions (Scheme 10). When subjecting the aryl‐S^VI^ oxytrifluoride **31** to BF_3_, oxocationic species **32** could be obtained, similar to the difluoride analogues (Scheme 3).^[18]^ However, structural evidence was not provided.

**Scheme 11:**
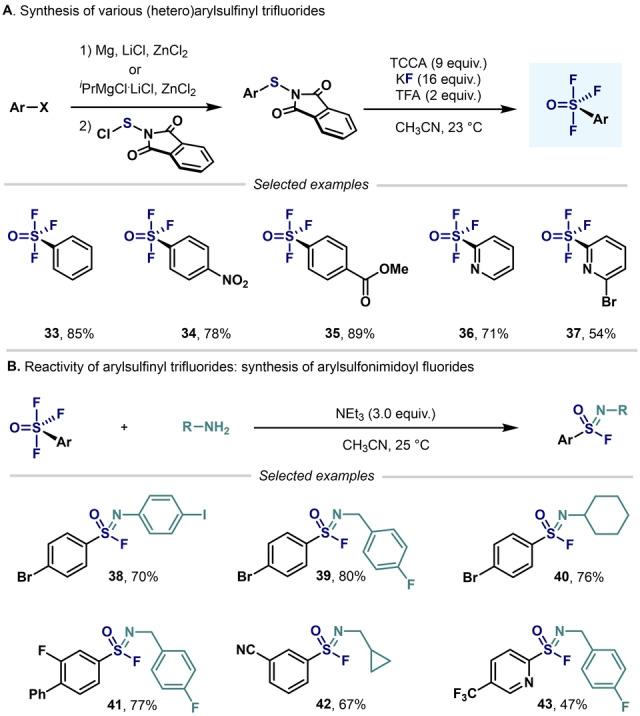
Synthetic procedure for ArSOF_3_ developed by Wang and Cornella.^[28]^

An advance in the area arrived in 2020, when Wang and Cornella developed a new method for the synthesis of ArSOF_3_ compounds (Scheme 11).^[28]^ This method provided a safer and more general platform to access ArSOF_3_, with excellent yields obtained for a wide range of compounds. This method capitalizes on the oxidative fluorination of Ar‐S(Phth) (generated in situ from aryl halides) to the corresponding ArSOF_3_ using easy‐to‐handle reagents and mild reaction conditions. Such compounds exhibit extremely high reactivity and rapidly evolve into their corresponding arylsulfonyl fluoride analogues (ArSO_2_F) upon exposure to trace amounts of H_2_O. The high sensitivity of the compounds made all efforts to isolate them unsuccessful. However, the high electrophilicity of ArSOF_3_ was turned into an advantage, and a wide range of primary aryl‐ and alkylamines were engaged in good yields, thus delivering highly coveted arylsulfonimidoyl fluorides.

## 4 Fluorination Level 4

**Scheme 12:**
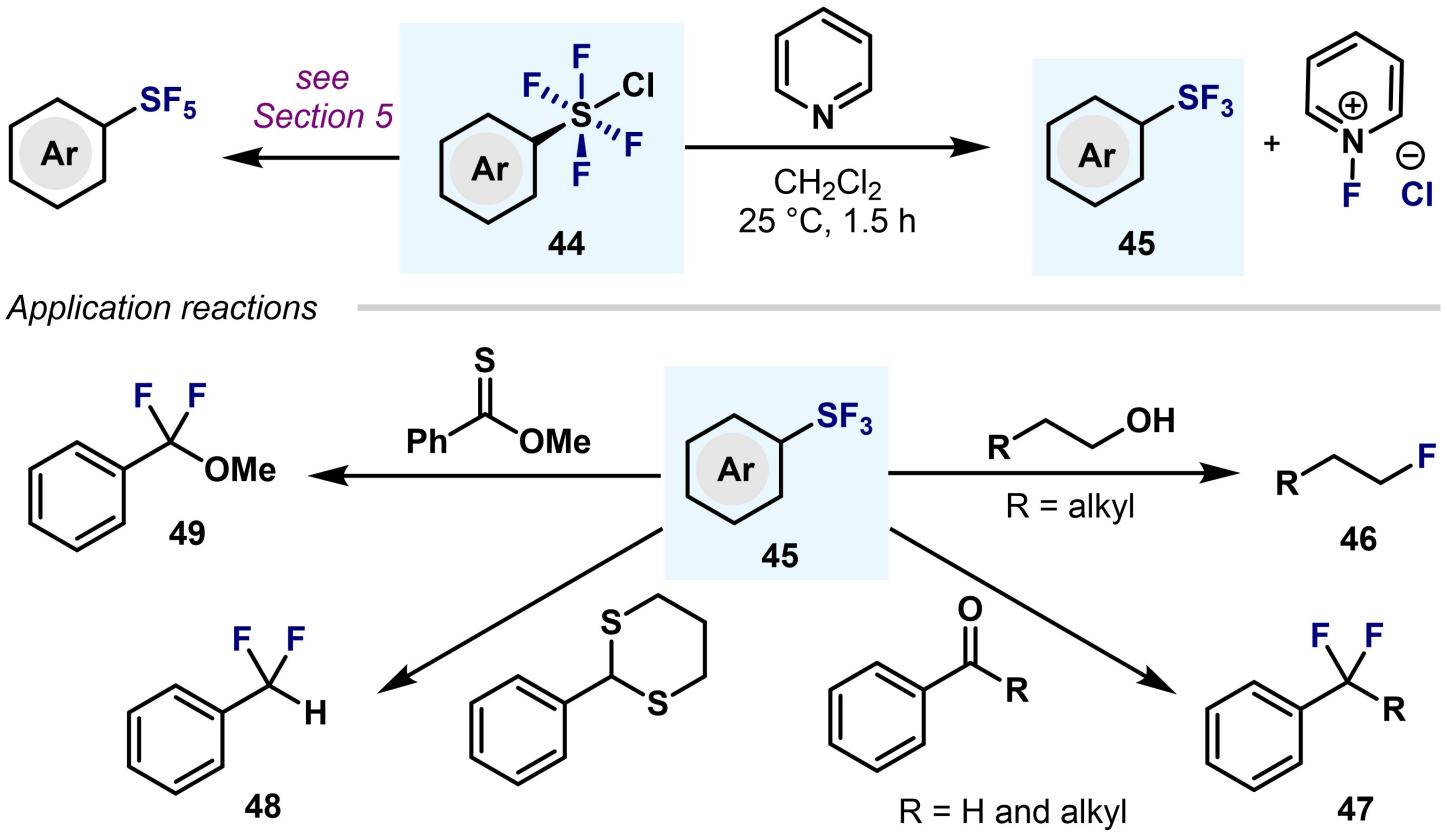
Applications of Ar−SF_4_Cl in organic synthesis.

The synthesis of aryl‐S^VI^ chlorotetrafluorides (Ar−SF_4_Cl) has been widely studied due to their potential subsequent applications. Ar−SF_4_Cl compounds are not only the most commonly utilized precursors to access level 5 (see Section 5), but also widely applied as deoxy‐ and desulfafluorinating agents (Scheme 12).^[29]^ Their high reactivity towards deoxyfluorination makes these reagents a good alternative to deoxyfluorinating agents such as SF_4_,^[30]^ DAST (diethylaminosulfur trifluoride),^[31]^ PhenoFluor [1,3‐bis(2,6‐diisopropylphenyl)2,2‐difluoro‐2,3‐dihydro‐1*H*‐imidazole],^[32]^ CpFluor (3,3‐difluoro‐1,2‐diarylcyclopropene),^[33]^ Fluolead (4‐*tert*‐butyl‐2,6‐dimethylphenylsulfur trifluoride),^[34]^ and PyFluor (2‐pyridinesulfonylfluoride),^[35]^ among others. Tetrafluoro‐λ^6^‐sulfanyl chlorides **44** do not react with C−O and C−S bonds; however, upon activation with a reductant such as pyridine, ArSF_3_ **45** is generated in situ and acts as a highly effective reagent for the deoxyfluorination of alcohols, aldehydes, ketones, carboxylic acids, and sulfur compounds (Scheme 12).

**Scheme 13:**
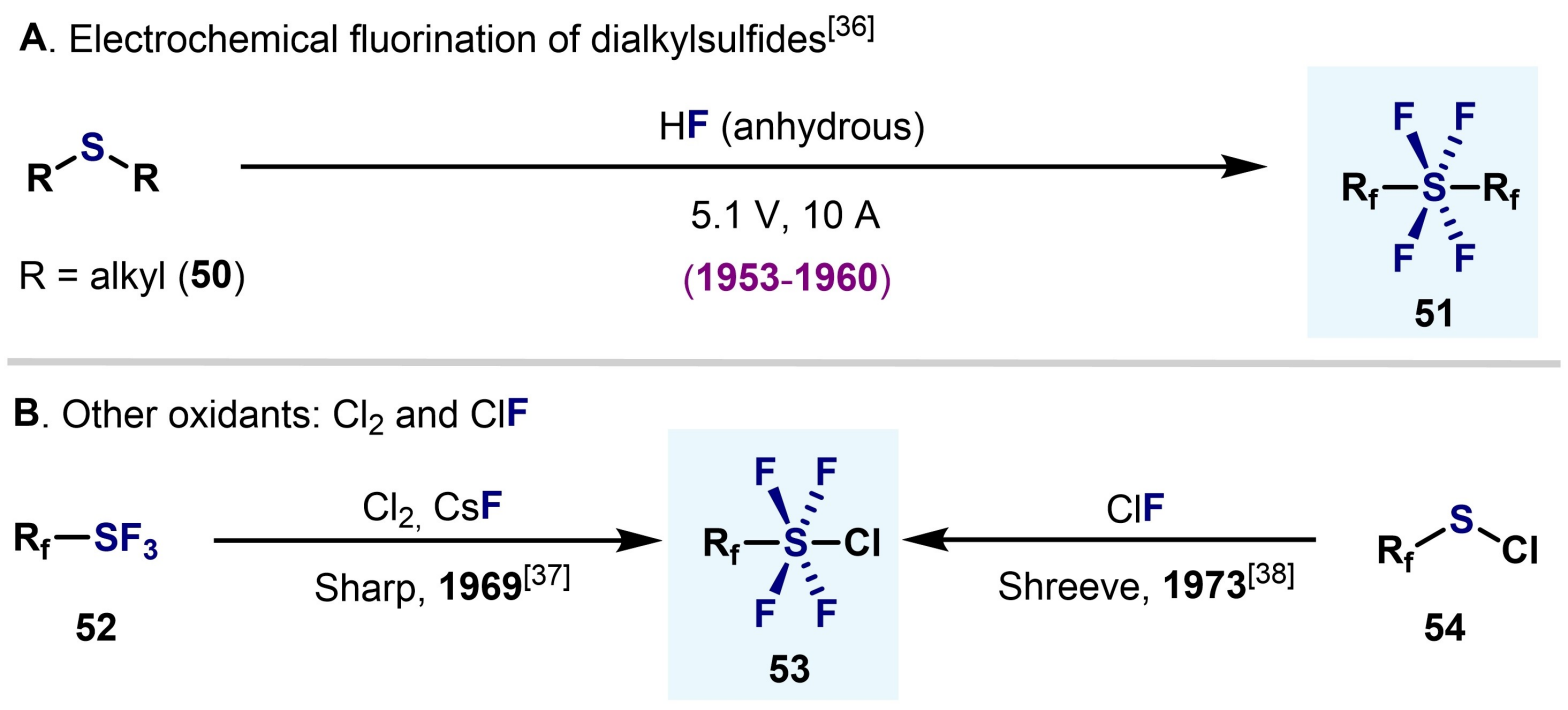
Early examples of perfluoroalkyltetrafluorosulfanes.

Early in the 1950s, aryl‐S^VI^ tetrafluorides were observed in crude mixtures from reactions between organosulfur(II) compounds and anhydrous hydrogen fluoride under electrolytic conditions.^[36]^ The harsh conditions resulted in low‐yielding mixtures of perfluorinated species (Scheme 13A). Years later, the groups of Sharp^[37]^ and Shreeve^[38]^ independently developed strategies to afford perfluoroaryl‐SF_4_Cl compounds, by using Cl_2_ or ClF as oxidants (Scheme 13B).

**Scheme 14:**
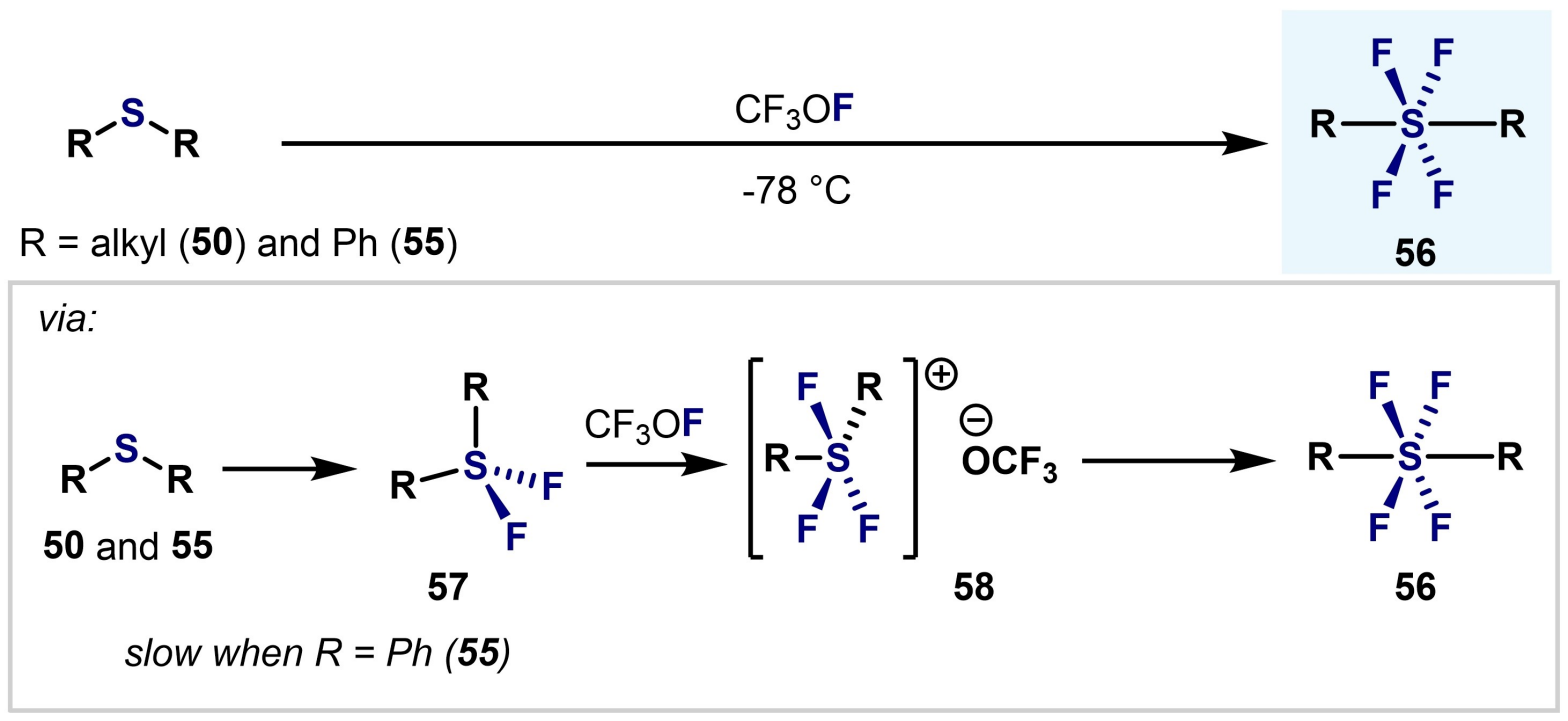
Early examples of alkyl‐ and aryltetrafluorosulfanes by Denney et al.^[39]^

It was in 1973 when Denney et al. developed a method for the synthesis of diaryl‐S^VI^ tetrafluorides using CF_3_OF as the oxidant (Scheme 14).^[39]^ Whereas dialkyl sulfides **50** reacted quickly at low temperatures, diphenylsulfide (**55**) required the presence of a large excess of CF_3_OF. Based on ^19^F NMR spectroscopy results, it was proposed that after an initial oxidation to the corresponding diaryl‐S(IV) difluoride **57**, cationic intermediate **58** forms, which eventually leads to **56** upon warming.

**Scheme 15:**
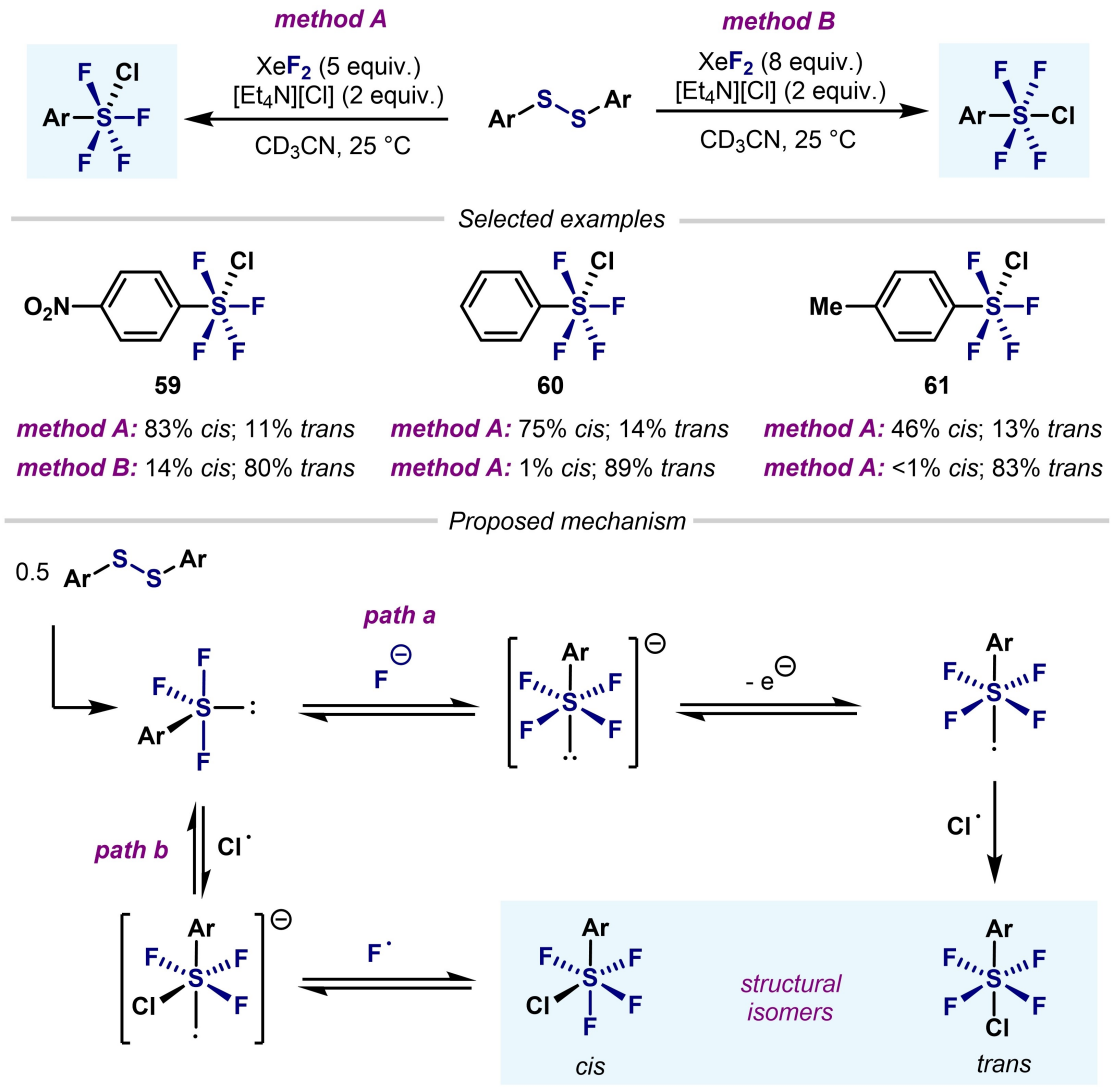
Synthesis of Ar−SF_4_Cl by Janzen and co‐workers.^[40]^

A breakthrough in the synthesis of Ar−SF_4_Cl arrived in 1997 when Janzen and co‐workers reported the oxidation of aryldisulfides with XeF_2_ in the presence of [Et_4_N][Cl] (Scheme 15).^[40]^ A large excess of XeF_2_ favored the formation of the *trans* isomer (method A), whereas lower amounts of oxidant favored the *cis* isomer (method B). Regardless of the method employed, analytically pure stereoisomers could not be obtained.

It was proposed that after oxidation of the disulfide, Ar−SF_3_ species are formed. At this point, two pathways can be envisaged. As already reported by Janzen and Ou,[^22^, ^23^] chlorine radicals (or Cl_2_) and fluoride anions are formed when XeF_2_ is mixed with chloride anions. Therefore, in path a (Scheme 15), fluoride anions react with Lewis acidic Ar−SF_3_, and after oxidation and radical coupling with a chlorine radical, the *trans* isomer is formed. On the other hand, Ar−SF_3_ can initially react with a chlorine radical (Scheme 15, path b) followed by radical coupling with a fluorine radical, thereby leading to the *cis* isomer. It was observed that over time, the *trans* isomer isomerizes to the *cis* isomer under the reaction conditions. Unfortunately, the authors did not provide an explanation for the effect of the Cl anions and excess XeF_2_ in the slow *trans*‐to‐*cis* isomerization.

**Scheme 16:**
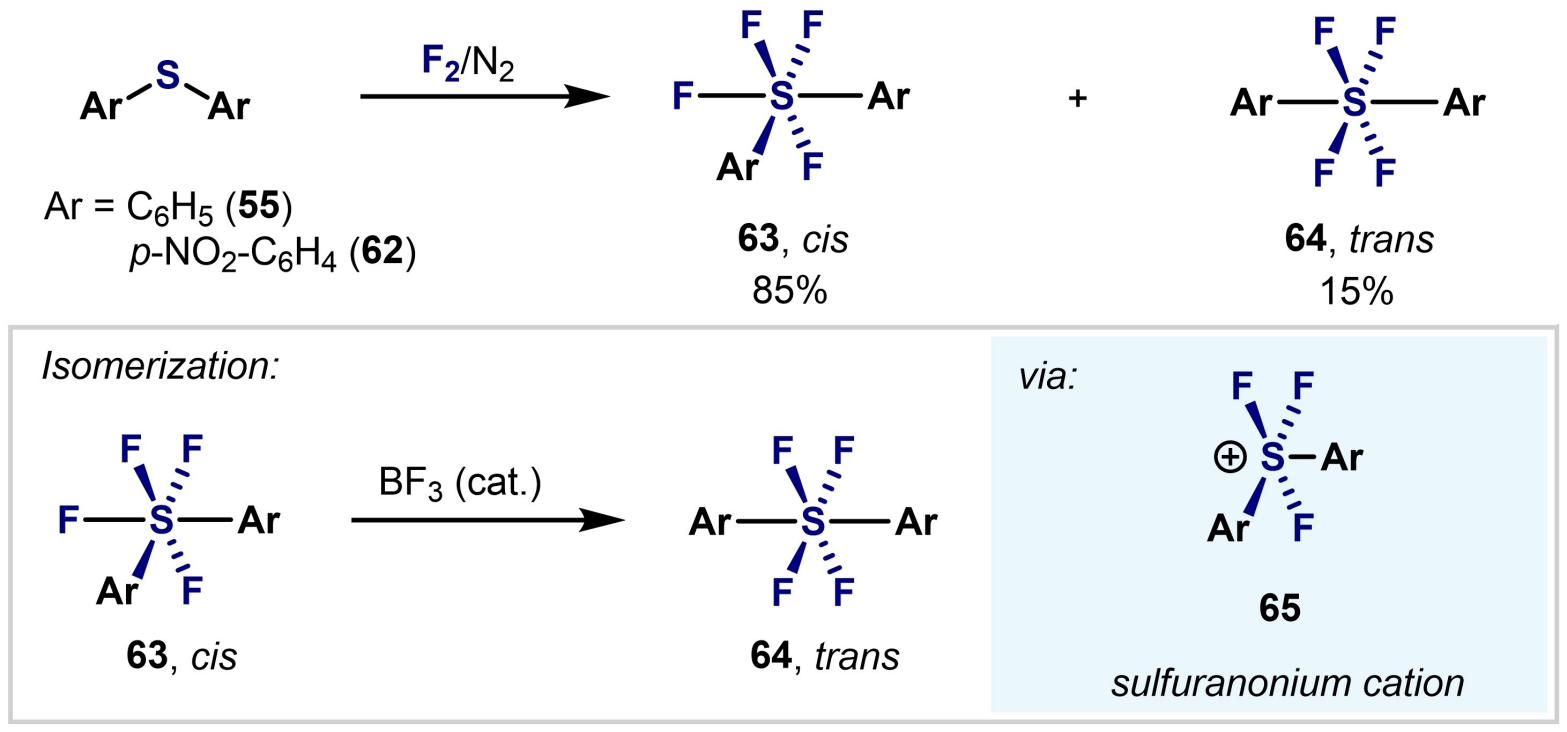
Synthesis of *cis*‐ and *trans*‐diaryltetrafluorosulfane mixture reported by Kirsch et al.^[41]^

Kirsch et al. reported the direct fluorination of diarylsulfides using F_2_/N_2_, which produced a mixture of *cis* and *trans* Ar_2_SF_4_ isomers (Scheme 16).^[41]^ Importantly, single‐crystal XRD structures were obtained for both isomers. Ab initio and DFT calculations suggested that a *cis*‐to‐*trans* isomerization can occur from a BF_3_‐based catalytic process via a sulfuranonium cation intermediate **65**, thus precluding thermal isomerization.

**Scheme 17:**
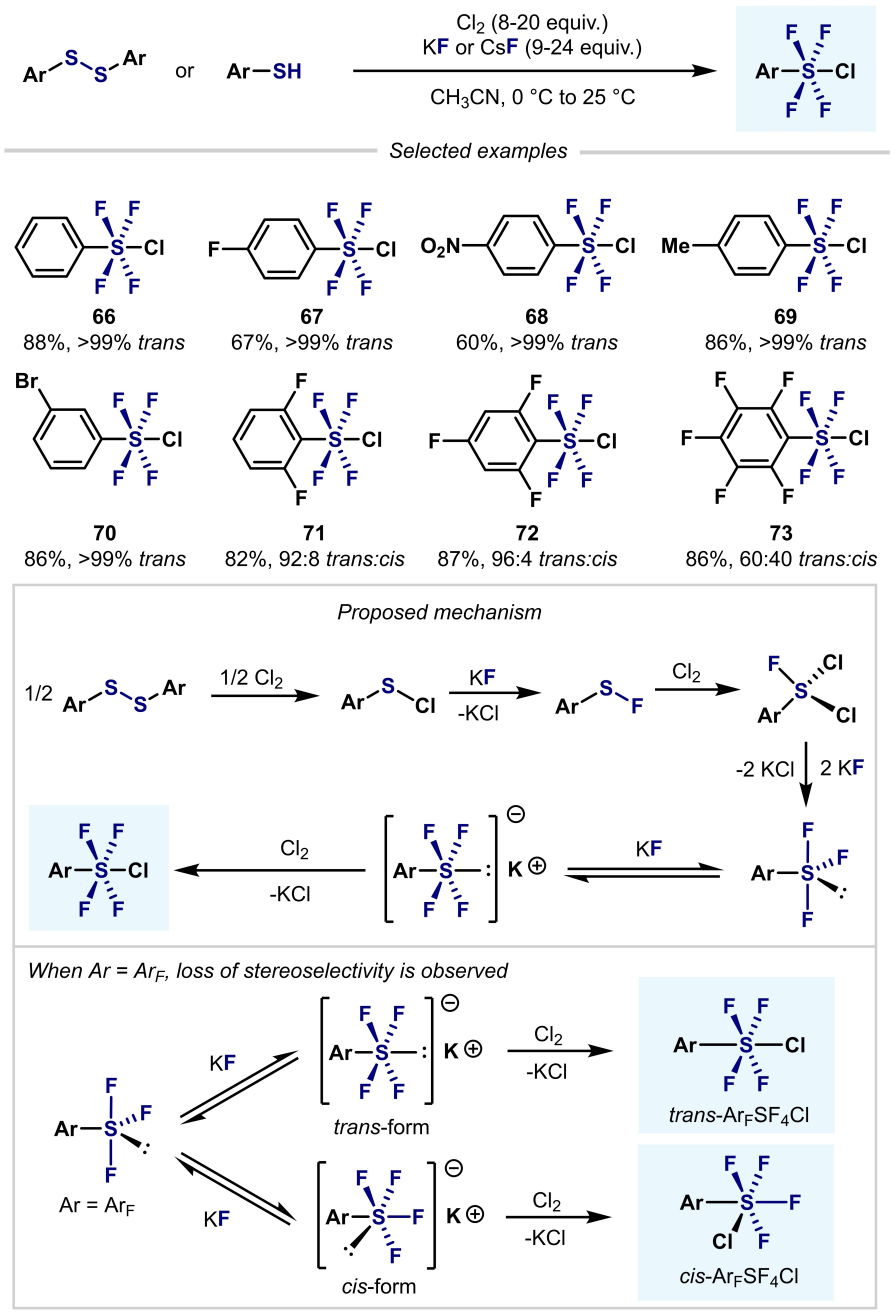
Synthesis of *trans*‐Ar−SF_4_Cl by Umemoto et al.^[42]^

An advance in this area was reported in 2012 by Umemoto et al. (Scheme 17),^[42]^ who used mild reaction conditions and a Cl_2_/KF system to convert a wide range of aryldisulfides or arylthiols into the corresponding aryltetrafluoro‐λ^6^‐sulfanyl chlorides in excellent yields and *trans* selectivity. Interestingly, when polyfluoroaryl‐SF_4_Cl was synthesized, mixtures of *trans* and *cis* isomers were obtained. Since thermal isomerization did not occur over time, the authors concluded that each isomer was formed through each isomeric salt (*trans* and *cis* form).

**Scheme 18:**
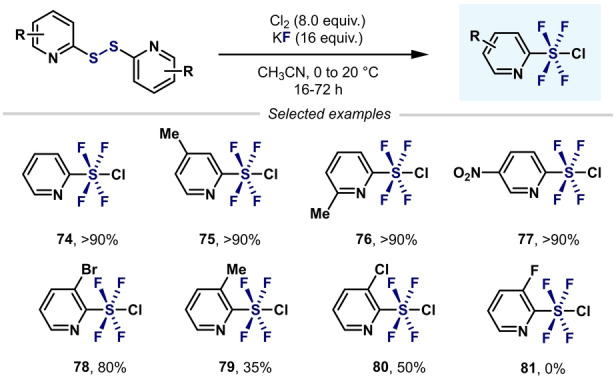
Synthesis of 2‐pyridyl‐SF_4_Cl by Kanishchev and Dolbier.^[43]^

Inspired by the procedure developed by Umemoto et al., Kanishchev and Dolbier and co‐workers synthesized a wide range of 2‐pyridyl‐S^VI^ chlorotetrafluorides with excellent *trans* stereoselectivity (Scheme 18).^[43]^ However, the presence of *ortho* substituents (F, Me, and Cl) to the S atom decreased the yield considerably (**79**–**81**), with the corresponding heteroaryl‐SF_3_ compounds afforded as side products in almost all cases.

**Scheme 19:**
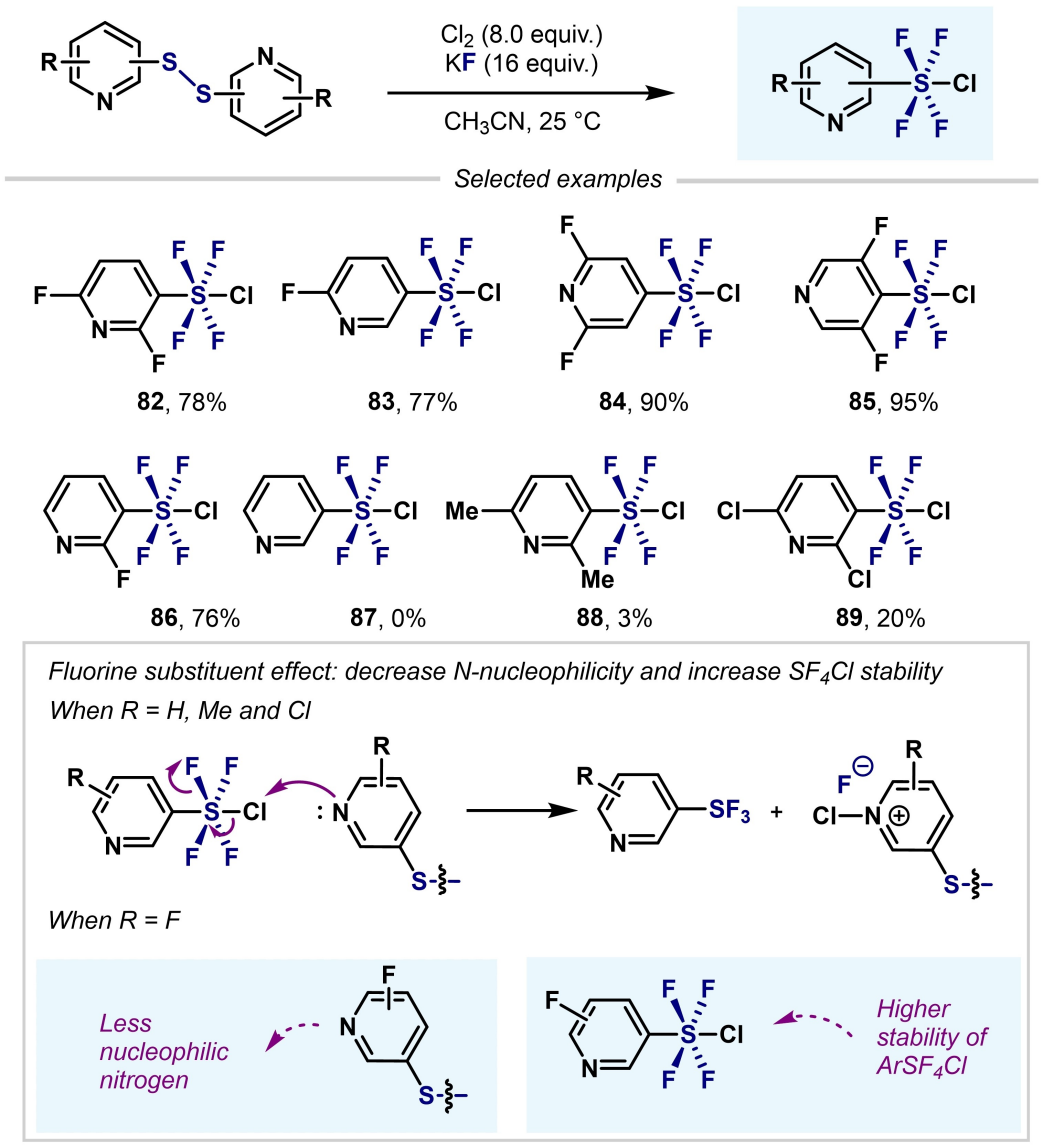
Effect of a fluorine substituent on the synthesis of pyridyl‐SF_4_Cl.^[44]^

Shibata and co‐workers expanded this reactivity to 3‐ and 4‐pyridyl‐SF_4_Cl (Scheme 19).^[44]^ The presence of fluorine atoms on the pyridine ring is essential for the successful conversion of pyridyldisulfides into the corresponding *meta*‐ and *para*‐aryl‐SF_4_Cl compounds. The presence of another substituent (H, Me, or Cl) on the pyridine moiety (**87**–**89**) led to the undesired heteroaryl‐SF_3_ being obtained. The authors hypothesized that the presence of an electron‐withdrawing group (EWG) such as fluorine would decrease the nucleophilicity of the N atom in the pyridine ring and, hence, stabilize the SF_4_Cl group.

**Scheme 20:**
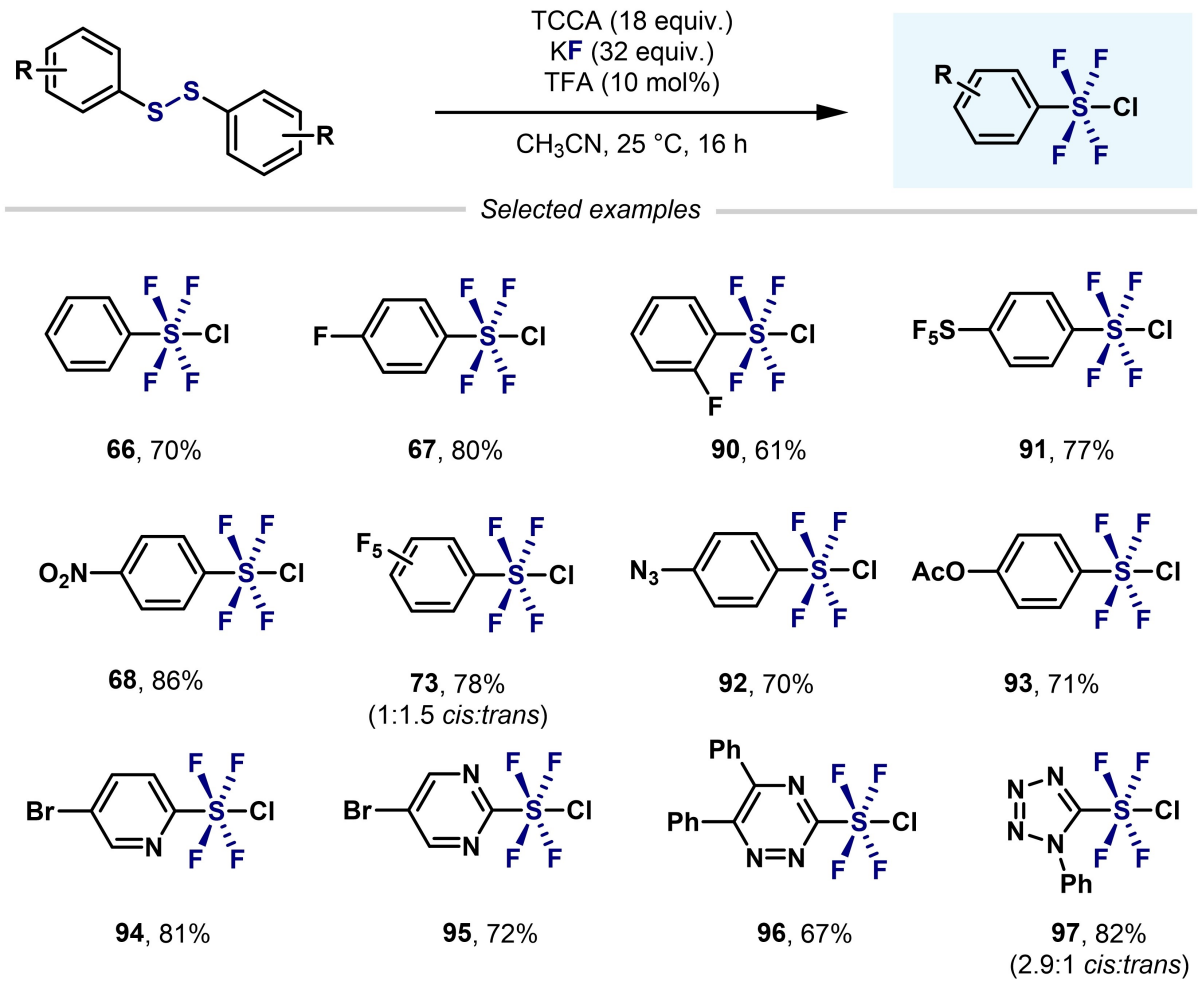
Synthesis of aryl‐ and heteroaryl‐SF_4_Cl developed by Pitts, Togni, Santschi et al..^[45]^

A remarkable breakthrough in this topic came in 2019 when Togni, Santschi, Pitts, and co‐workers presented the first approach to aryl‐SF_4_Cl that avoided the use of hazardous, gaseous oxidizing agents (e.g. F_2_ and Cl_2_). The method featured the easy‐to‐handle solid trichloroisocyanuric acid (TCCA), potassium fluoride (KF), and catalytic amounts of trifluoroacetic acid (TFA). This simple synthetic method permitted the synthesis of a wide range of aryl‐ and heteroaryl‐SF_4_Cl compounds in good yields (Scheme 20).^[45]^

**Scheme 21:**
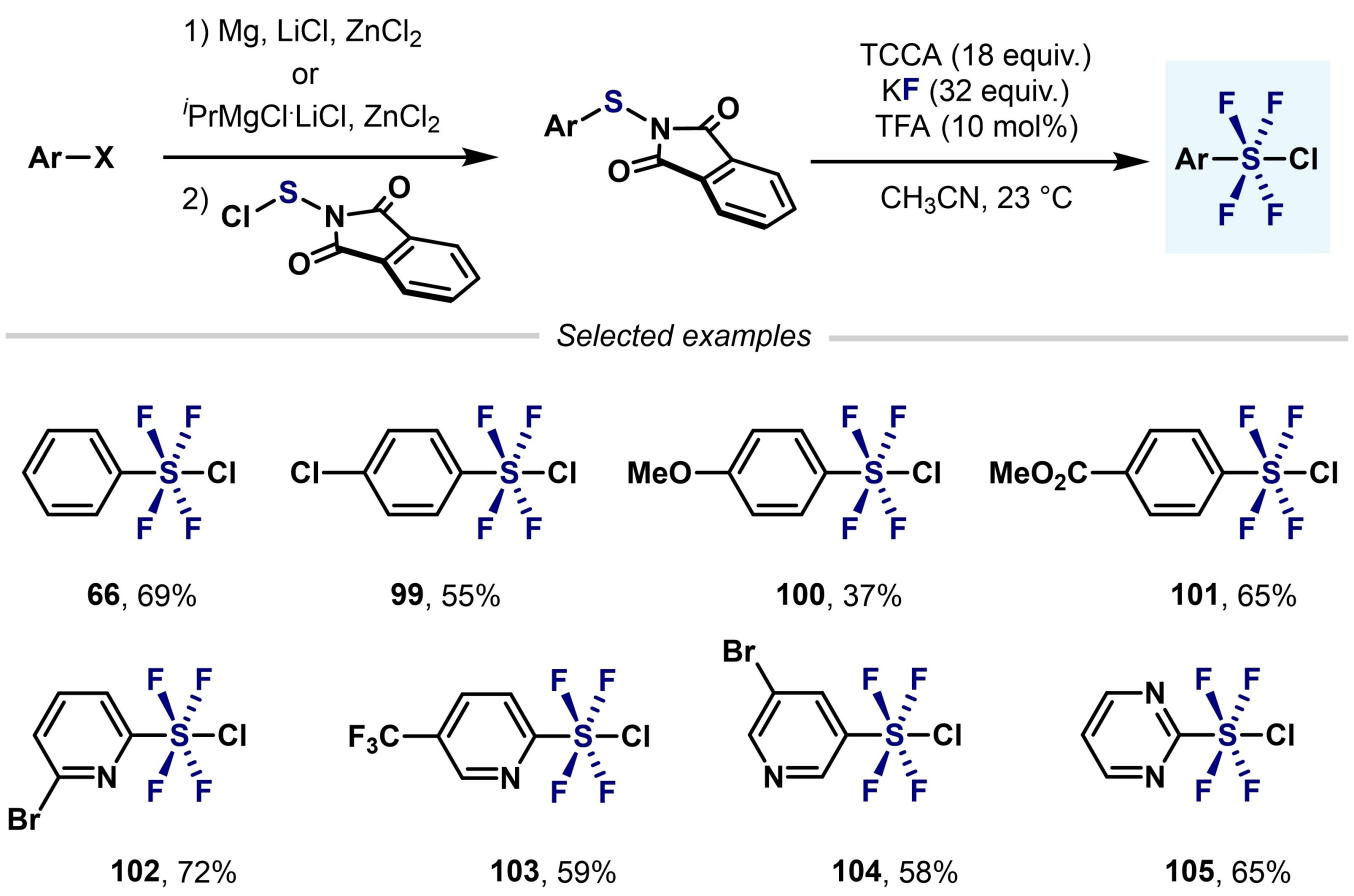
Synthesis of aryl‐ and heteroaryl‐SF_4_Cl from aryl halides developed by Wang and Cornella.^[28]^

Shibata and co‐workers used the method developed by Togni and co‐workers—without TFA—to obtain various (hetero)aryl‐SF_4_Cl (Scheme 21).^[46]^ However, longer reactions times were required.

**Scheme 22:**
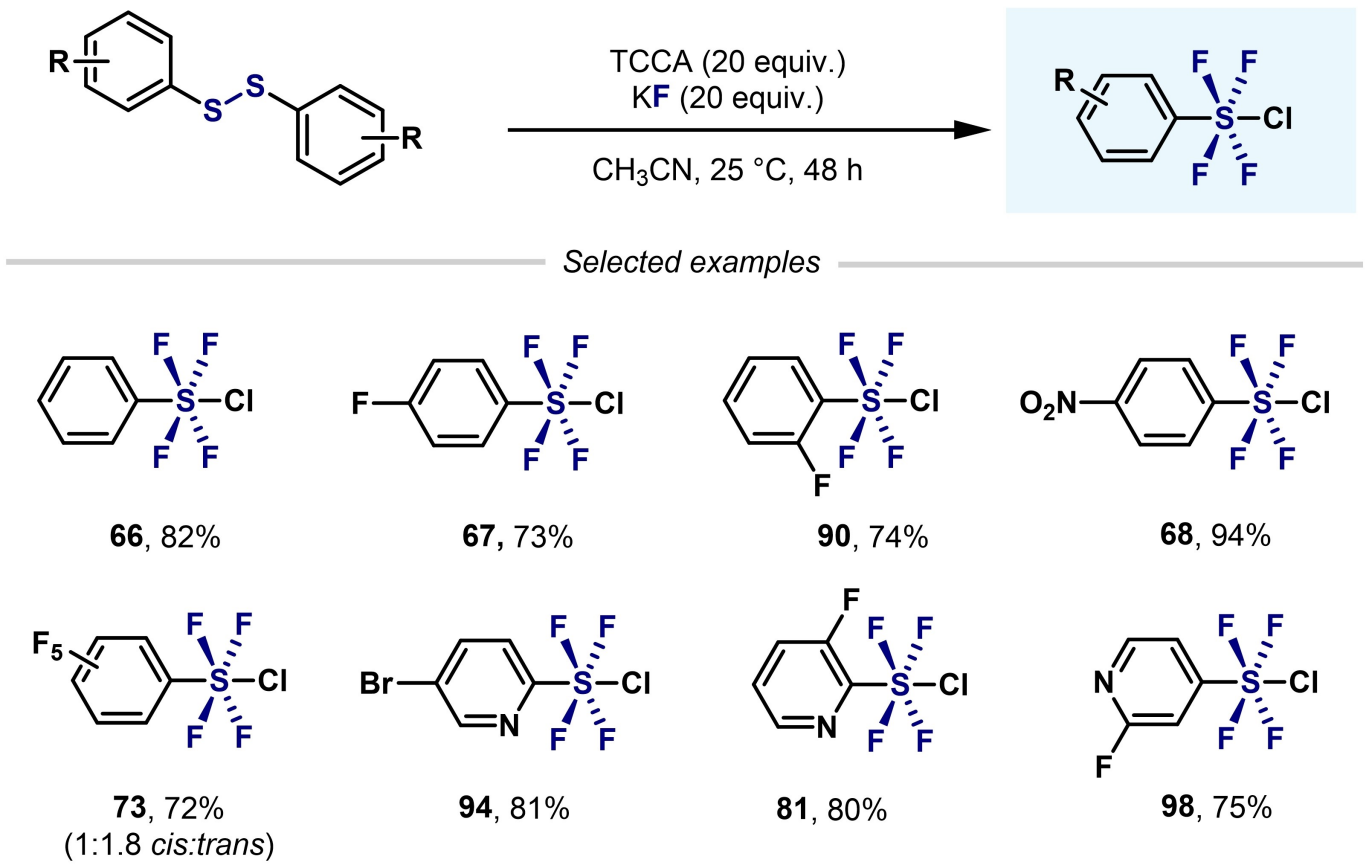
Synthesis of aryl‐ and (hetero)aryl‐SF_4_Cl developed by Shibata and co‐workers.^[46]^

Another improvement in the synthesis or aryl‐ and heteroaryl‐SF_4_Cl has recently been reported by Wang and Cornella (Scheme 22).^[28]^ Inspired by the synthetic method developed by Togni, Santschi, Pitts, and co‐workers, the authors were able to convert (hetero)aryl halides into the corresponding tetrafluoro‐λ^6^‐sulfanyl chlorides in excellent yields. It is important to highlight that, by slightly modifying the oxidation step, both level 3 (Scheme 11) and level 4 aryl‐S^VI^ fluorides could be obtained in excellent yields from the same Ar‐S(Phth) starting materials.

**Scheme 23:**
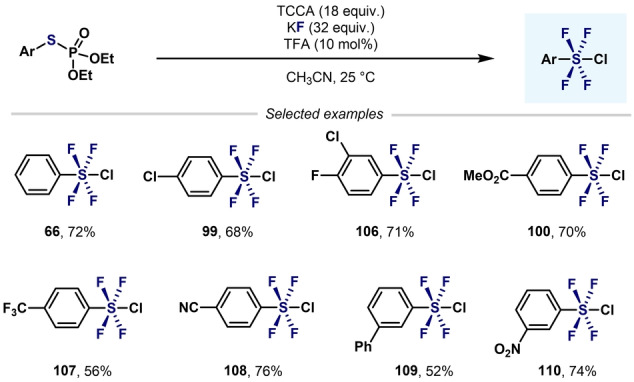
Synthesis of aryl‐SF_4_Cl from arylphosphorothiolates developed by Wang and Cornella.^[47]^

The same group, also reported the use of arylphosphorothiolates as convergent substrates for the synthesis of Ar−SF_4_Cl (Scheme 23).^[47]^ In this regard, similar yields were obtained as with the previous procedure using Ar‐S(Phth) (Scheme 22) as starting materials.^[28]^

Recently, Pascali and co‐workers reported a strategy to obtain aryltetrafluoro‐λ^6^‐sulfanyl chlorides by flow microfluidic technology.^[48]^ Unfortunately, this preliminary study only provided the Ar−SF_4_Cl compounds in low yields (5–10 %) together with undesired compounds such as ArSO_2_F and ArSOF.

**Scheme 24:**
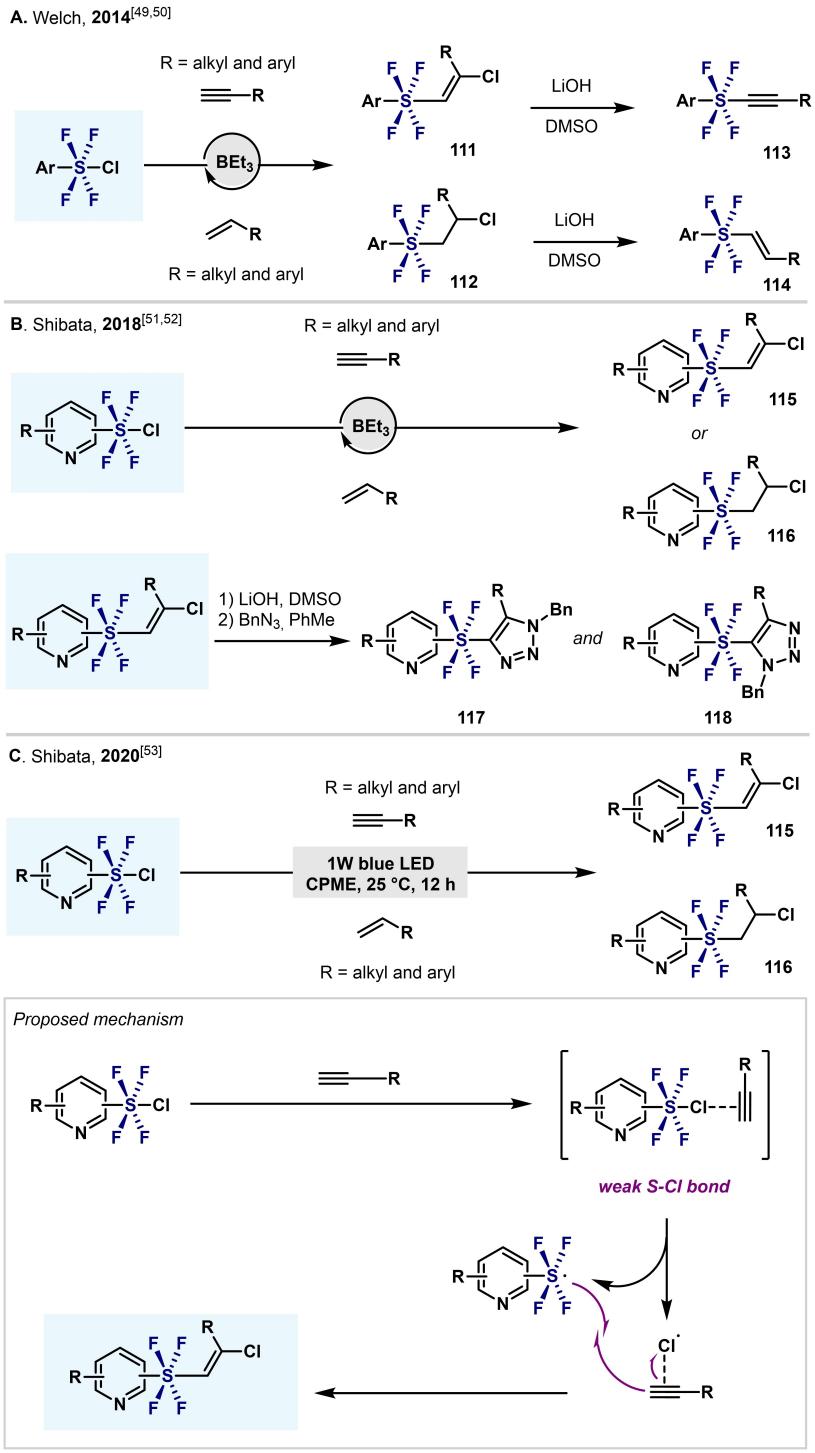
Application of Ar−SF_4_Cl in the synthesis of alkyl‐ and alkenyltetrafluoro‐λ^6^‐sulfanes and their derivatization.

As mentioned before (Scheme 12), (hetero)aryltetrafluoro‐λ^6^‐sulfanyl chlorides have attracted great attention as precursors of (hetero)aryl‐SF_5_ and as deoxyfluorinating agents.^[29]^ However, Ar−SF_4_Cl compounds have also been utilized in the synthesis of alkynyl‐ and alkenyl‐aryltetrafluoro‐λ^6^‐sulfanes, by capitalizing on the labile S−Cl bond (Scheme 24).

In 2014, Welch and co‐workers developed a BEt_3_‐catalyzed direct addition of Ar−SF_4_Cl to primary alkynes and alkenes through a S−Cl homolytic cleavage (Scheme 24A).[^49^, ^50^] Single‐crystal XRD revealed an octahedral geometry at the S atom, with all the fluorine atoms in the axial positions.^[49]^ Moreover, dehydrochlorination of the addition products (**111**, **112**) with lithium hydroxide formed the alkynyl **113** and (*E*)‐alkenyl‐aryltetrafluoro‐λ^6^‐sulfanes **114** in excellent yields, with no decomposition of the SF_4_ group. In 2018, Shibata and co‐workers synthesized pyridyltetrafluoro‐λ^6^‐sulfanes with alkenyl **115** or alkyl **116** substituents through a radical addition of pyridine‐SF_4_Cl to terminal alkynes and alkenes (Scheme 24B).^[51]^ By means of single‐crystal XRD and DFT calculations, the authors disclosed an octahedral geometry with a *trans* configuration of the hypervalent S^VI^ center. Furthermore, the *trans*‐tetrafluoro‐λ^6^‐sulfanes bearing an alkenyl group **115** were further derivatized through a thermal Huisgen 1,3‐dipolar cycloaddition to provide a wide range of three‐dimensional building blocks with two independent N‐heterocycles (**117**, **118**).^[52]^

In 2020 Shibata and co‐workers also reported the addition of Py‐SF_4_Cl to terminal alkynes and alkenes under irradiation with light (1 W blue LED; Scheme 24C). This procedure is an excellent alternative to BEt_3_‐catalyzed processes, as the borane is often the source of undesired side reactivity or substrate decomposition. In agreement with previous reports, the authors proposed a radical process to explain the reactivity observed.^[53]^

## 5 Fluorination Level 5

**Scheme 25:**
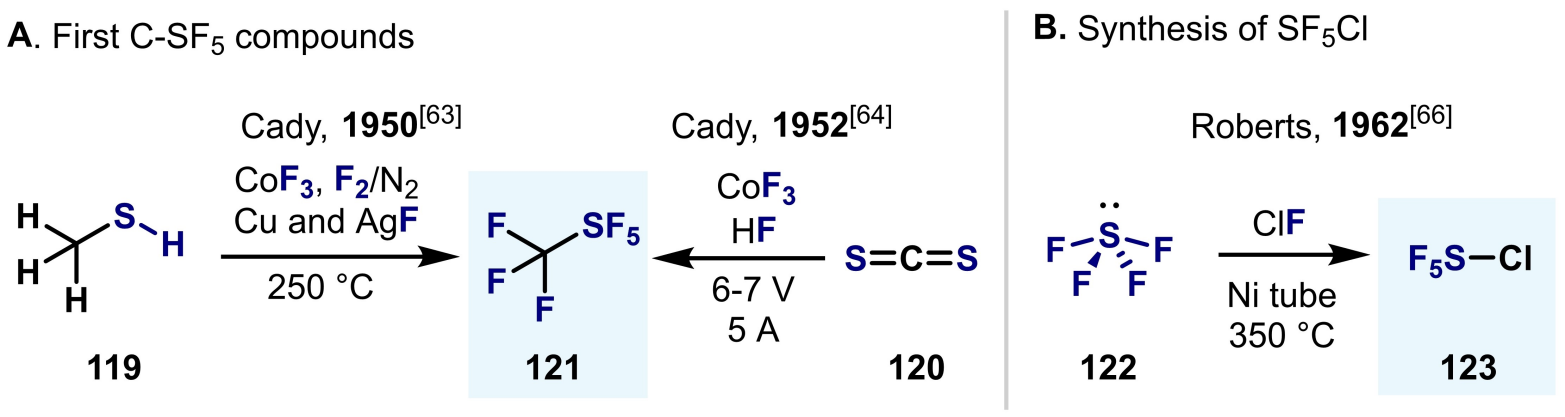
Pioneering syntheses of C−SF_5_ and SF_5_Cl.

In this level, only one type of compound reigns sovereign: namely, Ar−SF_5_. Although known for many years, it was only recently that the pentafluorosulfanyl group (SF_5_) became an interesting fluorine‐containing building block because of its thermal and chemical stability^[54]^ as well as inertness under physiological conditions.^[55]^ The electrostatic surface presented by the SF_5_ moiety is comparable to that of CF_3_ and its electron‐withdrawing effect suggests that the effects of SF_5_ and CF_3_ groups are similar in magnitude.^[56]^ The electronegativity of the SF_5_ group has been measured to be as high as 3.65, compared to a value of 3.36 for the CF_3_ group.^[57]^ The SF_5_ group is the newest member of a short list of functional groups that possess both high electronegativity and high lipophilicity, two properties that are generally juxtaposed. As a result of such unique properties, aryl‐SF_5_ compounds have attracted attention, and synthetic efforts towards their preparation have been the focus of intensive research.^[11]^ Investigations on Ar−SF_5_ span from applications as ^*t*^Bu and CF_3_ isosteres in medicinal chemistry,[^10^, ^58^] optoelectronic materials,^[59]^ or agrochemicals,^[60]^ to their ability to function as push‐pull fluoro‐^[61]^ and choromophores.^[62]^ Before discussing Ar−SF_5_ compounds, it is important to mention that the first syntheses of C−SF_5_ compounds were reported in the 1950s by Cady and co‐workers (Scheme 25).[^63^, ^64^] The authors reported the conversion of either methylmercaptan (**119**) or carbon disulfide (**120**) into CF_3_‐SF_5_ (**121**) using CoF_3_ and F_2_ ^[63]^ or HF in an electrochemical setup.^[64]^ Although the yields and purity of the mixtures were low, these synthetic procedures truly opened the door to a new era of S^VI^ pentafluoride chemistry.^[65]^ Equally important is the synthesis of SF_5_Cl (**123**) reported by Nyman and Roberts by the direct oxidation of SF_4_ (**122**) with ClF.^[66]^ Nowadays, SF_5_Cl (**123**) gas has become a benchmark reagent for the synthesis of SF_5_ compounds, and efforts toward its practical usage are of great interest.^[67]^

The synthesis of arylsulfanyl pentafluorides (Ar−SF_5_) can be classified on the basis of the synthetic approach. Therefore, we have organized this section in three subsections.

### 5.1 Direct Oxidation of Diaryldisulfides or Arylthiols

**Scheme 26:**
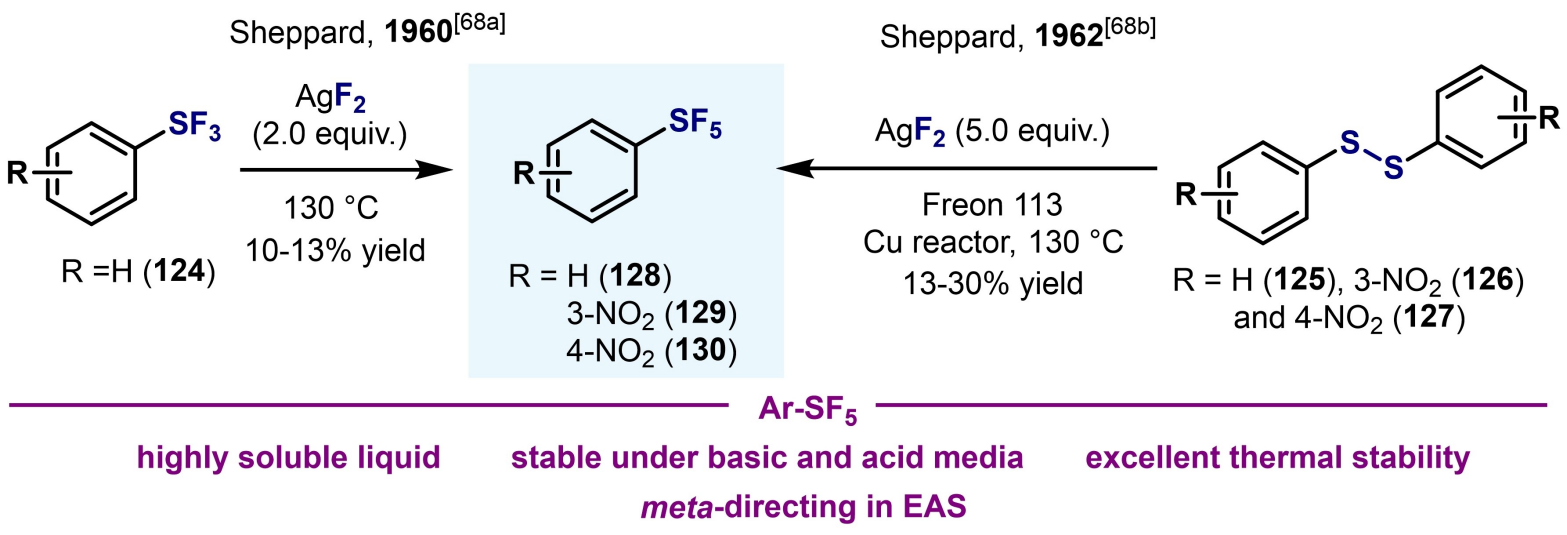
Early syntheses of Ar−SF_5_: synthesis and study of the physical and chemical properties by Sheppard.[^68^, ^69^]

The direct oxidation of diaryl disulfides or aryl thiols with strong fluorinating agents was the first approach toward the synthesis of arylsulfanyl pentafluorides (Ar−SF_5_).^[68]^ In all cases, low yields and narrow substrate scope were common features of those pioneering methods (Scheme 26). The first synthetic procedure for aryl‐SF_5_ compounds was reported by Sheppard et al. in 1960.^[68a]^ When phenylsulfur trifluoride (**124**, PhSF_3_) is heated gradually to 130 °C with AgF_2_ in a reactor made of copper or Teflon, phenylsulfur pentafluoride (**128**, Ph‐SF_5_) is obtained, albeit in low yields (10–13 %, Scheme 26).

Two years later, the same author slightly modified the previous procedure by adding aryl disulfides **125**–**127** to five molar equivalents of AgF_2_ suspended in 1,1,2‐trichloro‐1,2,2‐trifluoroethane (“Freon” 113) in a copper reactor.^[68b]^ This modified procedure was found to be particularly effective when NO_2_‐substituted Ar−SF_5_ **126** and **127** were utilized (Scheme 26). Several important conclusions arose from this work: 1) Ph‐SF_5_ is a colorless liquid that is soluble in common organic solvents, even hydrocarbons; 2) Ph‐SF_5_ shows excellent stability under basic conditions (NaOH) and acidic conditions (H_2_SO_4_), being hydrolyzed under the latter conditions only above 100 °C; 3) Ph‐SF_5_ also shows excellent thermal stability, even at 400 °C; 4) Ph‐SF_5_ directs nitration at the *meta*‐position; 5) the SF_5_ group is stable under catalytic hydrogenation conditions. Although low yields were obtained in all cases, this pioneering study from Sheppard et al. opened a new pathway in the synthesis or arylsulfur(VI) fluorides^[69]^ and the further application to several fields such as biology and medicinal chemistry.^[11]^

**Scheme 27:**
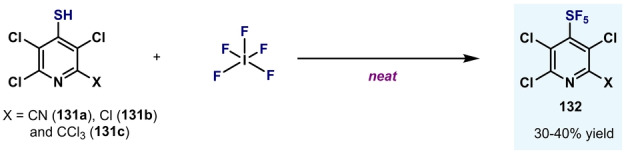
Other preliminary synthetic methods for the synthesis of Ar−SF_5_.^[70]^

Another direct oxidation procedure was developed by Karstev and co‐workers (Scheme 27),^[70]^ who synthesized polychloropyridine‐SF_5_ **132** by the direct oxidation of the corresponding thiols **131** with IF_5_. However, this procedure also suffered from low yields and narrow scope.

**Scheme 28:**
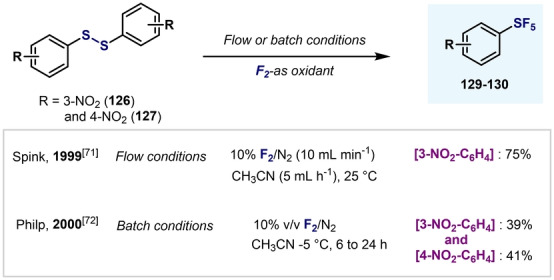
First examples of the synthesis of Ar−SF_5_ by direct oxidation using F_2_.

A few years later, the groups of Spink and Philp independently reported the use of F_2_ for the conversion of diaryldisulfides **126** and **127** into the corresponding Ar−SF_5_ compounds **129** and **130** (Scheme 28). Whereas Chambers and Spink developed the oxidation in flow conditions,^[71]^ Philp and co‐workers performed the oxidation in batch.^[72]^ Both methods led to improved yields and milder reaction conditions compared to Sheppard's AgF_2_‐based procedure.^[68]^

**Scheme 29:**
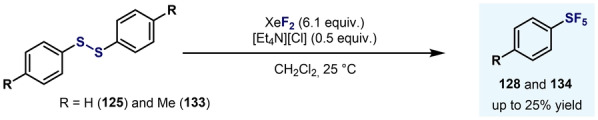
Synthesis of Ar−SF_5_ by direct oxidation using XeF_2_‐Et_4_NCl.^[23b]^

In 2000, Ou and Janzen applied the XeF_2_‐Et_4_NCl system to the conversion of a limited number of diaryldisulfides (**125** and **133**) into Ar−SF_5_ compounds (Scheme 29). In this case, low‐to‐moderate yields were obtained along with *trans*‐Ar−SF_4_Cl as the main by‐product.^[23b]^

**Scheme 30:**
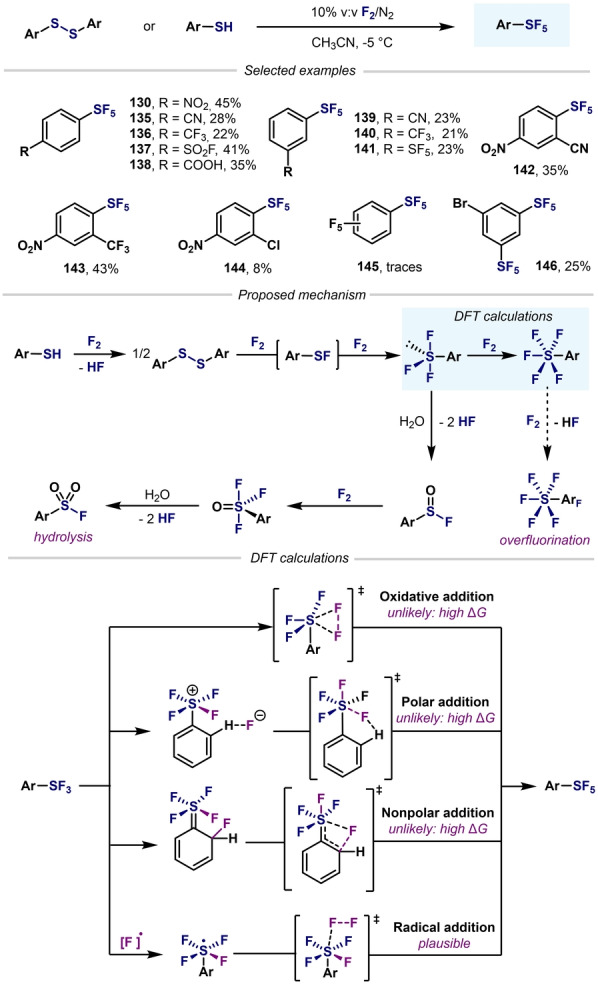
Synthesis of Ar−SF_5_ by direct oxidation using F_2_, as developed by Beier and co‐workers.^[73]^

In 2016 and 2019, Beier and co‐workers reported the direct fluorination of *ortho*‐, *meta*‐, and *para*‐substituted aromatic thiols and disulfides using F_2_ (Scheme 30).^[73]^ By comparing the synthetic performance under batch and flow conditions, it was found that a hybrid batch‐flow process (synthesis of Ar−SF_3_ in batch, then Ar−SF_5_ in flow) provided good yields.^[73b]^ By benchmarking experimental data with DFT calculations, the authors ruled out three nonradical pathways for the conversion of Ar−SF_3_ into Ar−SF_5_. It was finally proposed that the reaction proceeds through a radical mechanism after homolytic cleavage of the F−F bond. Propagation and termination steps are almost barrierless and the reaction depends on the stability of the Ar−SF_4_ radical species. However, further mechanistic insights are required to elucidate the mechanism for the direct fluorination of diaryldisulfides using F_2_.

### 5.2 Synthesis of Ar−SF_5_ from Ar−SF_4_Cl

**Scheme 31:**
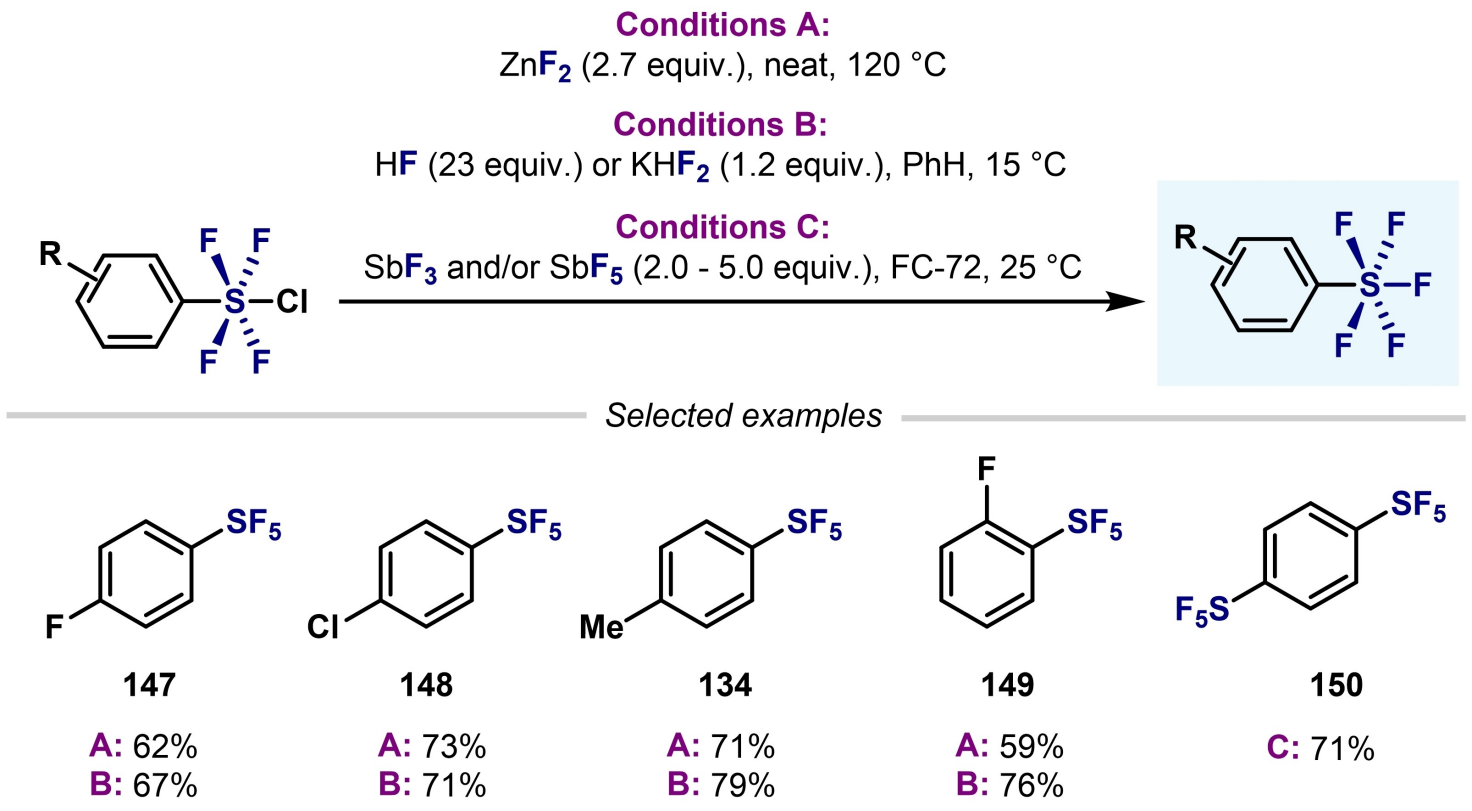
First synthesis of Ar−SF_5_ from Ar−SF_4_Cl by Umemoto et al.^[42]^

**Scheme 32:**
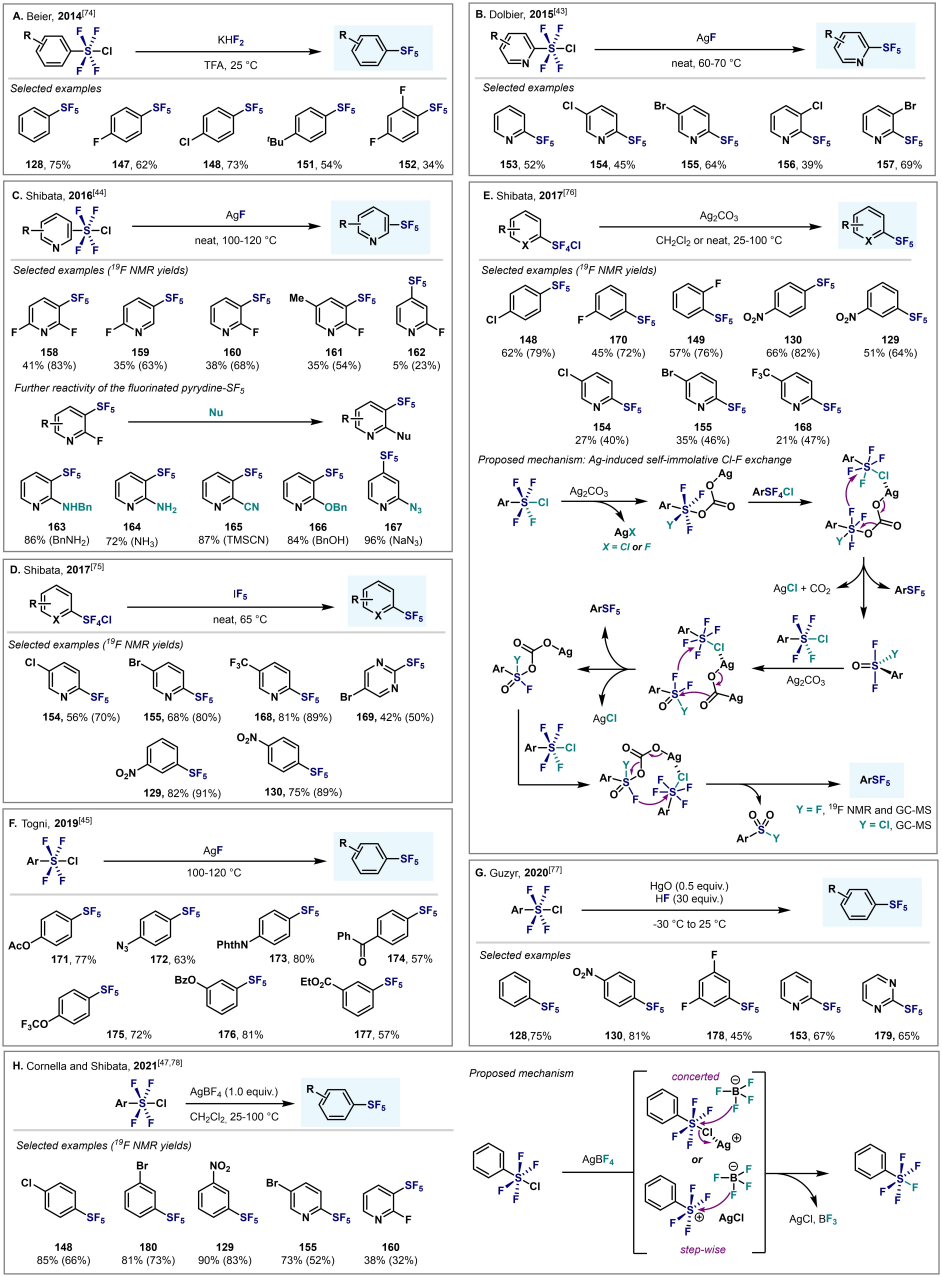
Conversion of Ar−SF_4_Cl into Ar−SF_5_: state‐of‐the‐art.

As mentioned in Section 4, Ar−SF_4_Cl species have been used as synthetic precursors of Ar−SF_5_ through Cl−F exchange. Compared to the direct fluorination of thiols or diaryldisulfides, the use of Ar−SF_4_Cl as precursors leads to higher yields of the desired Ar−SF_5_ under milder and safer conditions. In 2012, Umemoto et al. converted a wide range of Ar−SF_4_Cl into their corresponding Ar−SF_5_ compounds in good to excellent yields under mild reaction conditions, through a Cl–F exchange using either ZnF_2_‐HF (KHF_2_) or Sb^III/V^ fluorides (Scheme 31).^[42]^ Since then, several Cl–F exchange methods have been reported to expand the scope of possibilities to access Ar−SF_5_, and accommodate more functional groups (Scheme 32).

Beier and co‐workers reported that a combination of KHF_2_ and TFA at room temperature was optimal to convert various Ar−SF_4_Cl into their corresponding Ar−SF_5_ products (Scheme 32A).^[74]^ In this regard, comparable yields were obtained in almost all cases to those obtained with Umemoto's methodology. Kanishchev and Dolbier reported the conversion of 2‐pyridyl‐SF_4_Cl into the corresponding 2‐pyridyl−SF_5_ by using AgF as the fluoride source (Scheme 32B). These were pioneering examples of the efficient synthesis of highly sought after N‐heterocyclic−SF_5_ compounds.^[43]^ In 2016, Shibata and co‐workers reported a similar method for the conversion of a wide range of fluoro‐containing 3‐ and 4‐pyridyl−SF_4_Cl compounds, with moderate to good yields obtained (Scheme 32C).^[44]^ When treating 2‐fluoropyrdine‐SF_5_ compounds with different N‐ and O‐nucleophiles, nucleophilic aromatic substitution occurred, thus illustrating the great EWG ability of SF_5_. IF_5_ was also demonstrated to be a good fluoride source for the same purpose (Scheme 32D).^[75]^ Several years later, the same group reported a novel strategy for the synthesis of aryl‐ and heteroaryl‐S^VI^ pentafluorides by a Ag_2_CO_3_‐induced Cl–F exchange (Scheme 32E).^[76]^ Remarkably, this fluorination does not require any external fluoride sources; rather, the reaction proceeds through a self‐immolative mechanism of Ar−SF_4_Cl. In 2019, Togni and co‐workers also converted a wide variety of Ar−SF_4_Cl compounds into the corresponding Ar−SF_5_ derivatives by using AgF as the classic external fluoride source (Scheme 32F).^[45]^ Recently, Guzyr et al. reported the synthesis of aryl‐ and heteroaryl‐S^VI^ pentafluorides using HgO and HF (Scheme 32G).^[77]^ Very recently, the Cornella and Shibata groups independently showed that AgBF_4_ is also a valid source of F for the synthesis or Ar−SF_5_ compounds (Scheme 32H).[^47^, ^78^] The authors proposed activation of the Cl atom of (hetero)aryl‐SF_4_Cl by Ag^+^, and subsequent attack of the fluoride atom of the BF_4_ anion to the S center by either a concerted or stepwise mechanism.

### 5.3 Alternative Syntheses Using SF_5_Cl

**Scheme 33:**
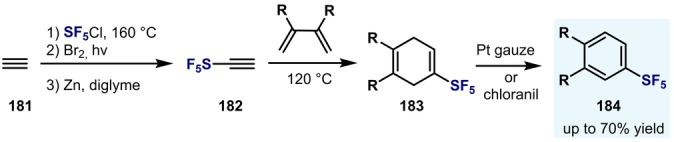
Synthesis of Ar−SF_5_ developed by Hoover and Coffmann.^[79]^

In Sections 5.1 and 5.2 it was shown that direct oxidative fluorination and F–Cl exchange from Ar−SF_4_Cl represents the standard approach for the synthesis of Ar−SF_5_ compounds. Nevertheless, other alternative synthetic procedures involving the de novo synthesis of the aromatic ring have appeared in the literature. For example, Ar−SF_5_ can be assembled through a Diels–Alder reaction of ethynylsulfur pentafluoride (**182**) with different dienes, as demonstrated by Hoover and Coffmann (Scheme 33).^[79]^

**Scheme 34:**
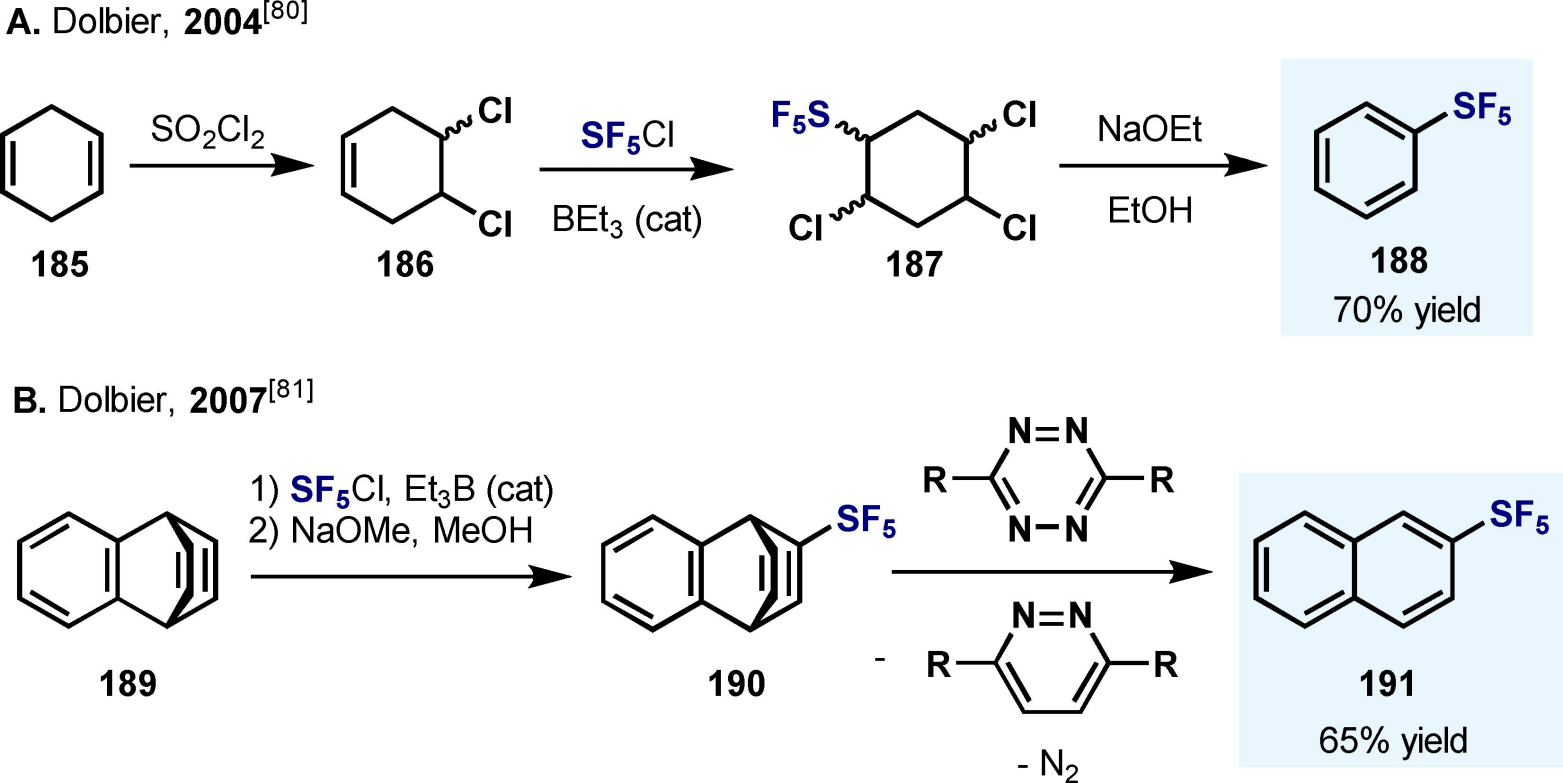
Synthesis of Ar−SF_5_ developed by Sergeeva and Dolbier.^[80]^

Sergeeva and Dolbier developed a convenient three‐step synthesis of Ph‐SF_5_ from 1,4‐cyclohexadiene (**165**) with an overall yield of 70 %. The key step in this synthesis is the radical addition of SF_5_Cl to an alkene, thereby forging a C−SF_5_ bond in almost quantitative yield (Scheme 34A).^[80]^

The same group also developed a three‐step synthesis of 2‐pentafluorosulfanylnaphthalene **(191**) by the initial addition of SF_5_Cl to benzobarralene **189**. Elimination of the ethylene bridge by a cycloaddition/retro‐cycloaddition sequence with 3,6‐bis‐(2‐pyridyl)‐1,2,4,5‐tetrazine afforded the Ar−SF_5_ **191** in good yield (Scheme 34B).^[81]^

**Scheme 35:**
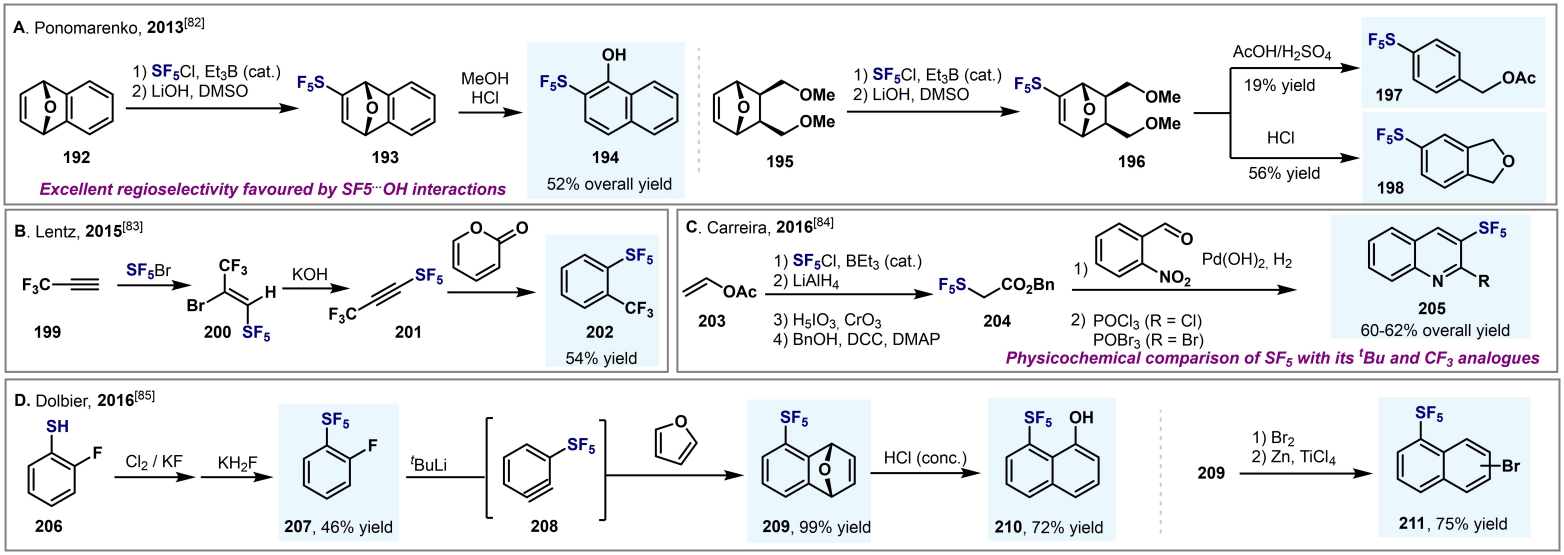
Synthesis of different aryl‐SF_5_ and heteroaryl‐SF_5_ compounds.

Ponomarenko et al. reported the Et_3_B‐catalyzed SF_5_Cl radical addition reactions of substituted aryl‐ and naphthyl‐SF_5_ from 7‐oxanorbornene derivatives **192** and **195** (Scheme 35A).^[82]^ The high regioselectivity observed for the formation of 2‐SF_5_‐1‐naphthol (**194**) is consistent with ab initio computational studies, which revealed a SF_5_⋅⋅⋅HO hydrogen bond that renders additional stabilization to the product. Duda and Lentz prepared pentafluoro(3,3,3‐trifluoroprop‐1‐yn‐1‐yl)‐λ^6^‐sulfane (**201**) in high yields in two steps from 3,3,3‐trifluoropropyne (**199**) by the addition of SF_5_Br followed by a dehydrobromination reaction (Scheme 35B).^[83]^ This compound was demonstrated to be a good dienophile in Diels–Alder reactions, as shown by its reaction with pyranone. Importantly, this procedure permits the introduction of the SF_5_ group at the *ortho* position of arenes (**202**). Carreira and co‐workers reported a synthetic strategy for preparing SF_5_‐containing N‐heterocyclic building blocks, such as quinolinones, quinolines, and pyridines (Scheme 35C).^[84]^ Benzyl SF_5_‐acetate (**204**) proved to be an excellent candidate to participate in aldol reactions, providing a wide range of 3‐SF_5_‐quinolinones in good to excellent yields. These compounds can be rapidly converted into the corresponding quinolines **205** using either POCl_3_ or POBr_3_.

Since the SF_5_ group is considered a bioisostere of the CF_3_ and ^*t*^Bu groups, the authors compared the physicochemical properties of 3‐SF_5_‐quinolinone with its CF_3_ and ^*t*^Bu analogues. Preliminary collected data showed that SF_5_ exhibits higher lipophilicity than CF_3_, but lower than the ^*t*^Bu group. The membrane permeability increases in the order SF_5_<^*t*^Bu<CF_3_. In terms of p*K*_a_ values, the SF_5_‐quinolinone was the most acidic. Concerning the solubility, both the CF_3_ and SF_5_ compounds are considerably more soluble than the ^*t*^Bu counterpart.

Kanishchev and Dolbier generated *ortho*‐SF_5_‐benzyne (**208**) by a lithiation/elimination sequence starting from 2‐fluoro‐SF_5_‐benzene **207** (Scheme 35D).^[85]^ The highly reactive SF_5_‐benzyne intermediate underwent Diels–Alder cyclization with furan in situ, and the product **209** was subjected to a series of further chemical transformations en route to **210** and **211**.

All these synthetic alternatives allow access to *ortho*‐substituted aryl‐SF_5_ compounds in good yields, in contrast to direct oxidative fluorination (Section 5.1), where the *ortho*‐substituted diaryldisulfides are still the main limitation to expand the substrate scope.

## 6 Conclusion and Outlook

This Minireview highlights different approaches toward the synthesis aryl‐S^VI^ fluorides, where the central S atom is substituted with 2, 3, 4, or 5 F atoms, thus defining the fluorination level. Level 2 fluorinated compounds have been successfully prepared by using XeF_2_/Et_3_NCl as an oxidative fluorinating system, a method that still remains in use nowadays. Remarkably, these compounds have gained much attention recently, as the corresponding sulfoxonium cations have been shown to be super‐Lewis acids for organic transformations.

Level 3 fluorination is still an underdeveloped platform, with untapped potential for synthesis. It is clear that ArSOF_3_ compounds are good linchpin reagents for the synthesis of sulfonimidoyl fluorides.

Whereas levels 2 and 3 are still underdeveloped, their higher fluorinated analogues (levels 4 and 5) have been widely studied and their synthesis widely explored. In this regard, recent advances in the use of Ar−SF_4_Cl compounds have shown that strong oxidants such as F_2_ and Cl_2_ can be replaced by the safer and easy‐to‐handle TCCA/KF system.

Finally, the synthesis of arylsulfanyl pentafluorides have been the most studied and improved, as a result of the recent interest of Ar−SF_5_ compounds in medicinal chemistry. The current synthetic approaches rely on the Cl–F exchange from the corresponding Ar−SF_4_Cl compounds. Alternatively, other synthetic procedures that employ SF_5_Cl gas as a precursor to the SF_5_ group have also been successful, although the synthesis of the aryl ring is required.

Despite the excellent advances in the synthesis of (hetero)arylsulfur(VI) fluorides and their successful applications, the reported approaches still suffer from several disadvantages, such as narrow substrate scope and harsh reaction conditions. Thus, we envision that innovative work in this area is likely to arise from new greener and milder synthetic methods. Moreover, as a result of the high interest in the SF_5_ group in medicinal chemistry and biology, this area will also evolve to avoid the use of toxic SF_5_Cl as a SF_5_ radical precursor, and the use of the greener, but less reactive, SF_6_ gas. The very few examples of this latter approach already show the promising synthetic utility of SF_6_ gas as a building block for sulfur(VI) fluoride synthesis.^[86]^

## Conflict of interest

The authors declare no conflict of interest.

## Biographical Information

*Marc Magre earned his PhD under the supervision of Prof. Montserrat Diéguez and Prof. Oscar Pàmies at Universitat Rovira i Virgili (Spain) in 2016, working on tailor‐made chiral Pd‐ and Ir‐based catalysts for enantioselective transformations. In 2017, he joined the group of Prof. Magnus Rueping (RWTH Aachen; Germany) as a postdoctoral researcher, where he developed novel magnesium‐catalyzed hydrofunctionalizations of unsaturated systems. In 2020, he joined the Max Planck Institut für Kohlenforschung (Mülheim an der Ruhr, Germany) as a postdoctoral researcher in the group of Dr. Josep Cornella, where he focuses on bismuth catalysis*.

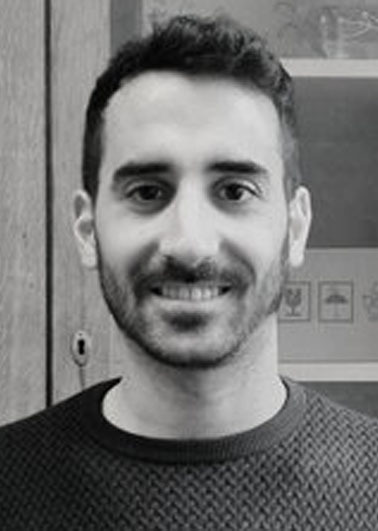

## Biographical Information

*Shengyang Ni was born in Yancheng (China), in 1992. He earned his PhD under the supervision of Prof. Yi Pan at Nanjing University (China) in 2019, working on Ni‐catalyzed reductive cross‐coupling reactions. During this time, he was a visiting PhD student (2017–2019) in the group of Prof. Phil Baran at Scripps Research Institute (US). After his PhD, he stayed in the same group to continue his postdoctoral research. In 2021, he joined the Max Planck Institut für Kohlenforschung (Mülheim an der Ruhr, Germany) as a postdoctoral researcher in the group of Dr. Josep Cornella, where he focuses on Ni‐catalyzed N_2_O activation*.

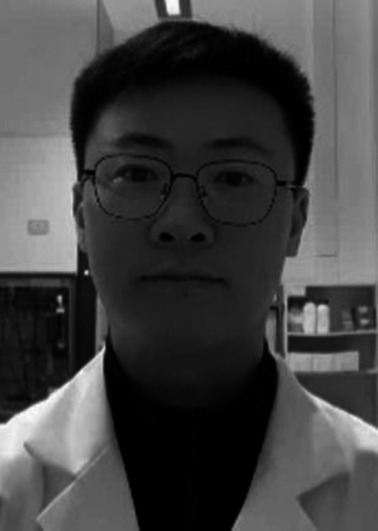

## Biographical Information

*Josep Cornella (Pep) studied chemistry at the University of Barcelona (2008) and completed his PhD in 2012 at Queen Mary University (UK) under the supervision of Prof. Igor Larrosa. He then pursued postdoctoral studies in the groups of Prof. Ruben Martin (ICIQ, Spain) as a Marie Curie Postdoctoral Fellow and Prof. Phil S. Baran (The Scripps Research Institute, California, USA) as a Beatriu de Pinos Fellow. In 2017, he was appointed as a Max Planck Research Group Leader in the Department of Organometallic Chemistry at the Max‐Planck‐Institut für Kohlenforschung in Mülheim an der Ruhr, Germany, where he leads the Sustainable Catalysis Laboratory*.